# Engineering of functional auditory neurons from human induced pluripotent stem cells

**DOI:** 10.1016/j.mmr.2026.100008

**Published:** 2026-04-15

**Authors:** Minjin Jeong, Lucas G. Vattino, Maja Djurisic, Kevin P. Rose, Hiroshi Hyakusoku, Svetolik Spasic, Xiao-Jie Ma, Hiroaki Mohri, Ronna Hertzano, Anne E. Takesian, Konstantina M. Stankovic

**Affiliations:** a Department of Otolaryngology-Head and Neck Surgery, Stanford University School of Medicine, Stanford, CA 94304, USA; b Department of Otolaryngology-Head and Neck Surgery, Massachusetts Eye and Ear and Harvard Medical School, Boston, MA 02114, USA; c Section on Omics and Translational Science of Hearing, Neurotology Branch, National Institute on Deafness and Other Communication Disorders, National Institutes of Health, Bethesda, MD 20892, USA; d Department of Otorhinolaryngology, Yokosuka Kyosai Hospital, Kanagawa 238-8558, Japan; e Department of Otolaryngology, Qilu Hospital of Shandong University, Jinan 250062, China; f Department of Neurosurgery, Stanford University School of Medicine, Stanford, CA 94304, USA; g Wu Tsai Neurosciences Institute, Stanford University, Stanford, CA 94304, USA

**Keywords:** Spiral ganglion neurons (SGNs), Human induced pluripotent stem cells (hiPSCs), Auditory neurons, Cochlea, Inner ear

## Abstract

**Background:**

Spiral ganglion neurons (SGNs) relay auditory sensory information from the cochlea to the brain. Their loss results in permanent hearing impairment in humans due to their limited regenerative capacity. Progress in hearing restoration has been constrained by the inaccessibility of human inner ear tissue and challenges in generating functionally mature human SGN-like neurons from stem cells *in vitro*.

**Methods:**

To generate human SGN-like neurons from human induced pluripotent stem cells (hiPSCs), we recapitulated key signaling pathways involved in human inner ear development. On day (D) 11 of differentiation, nerve growth factor receptor-positive cells (precursors of pre-placodal ectoderm and neural crest) were isolated using magnetic sorting. From D18 to D25, cultures were treated with sonic hedgehogs to induce otic neural progenitors. Neuronal maturation was subsequently promoted by a cocktail of brain-derived neurotrophic factor, neurotrophin-3, and insulin-like growth factor-1, which supports SGN development. Cellular identity and functionality were assessed using single-cell RNA sequencing, immunocytochemistry, whole-cell patch-clamp electrophysiology, co-culture assays, and calcium ion (Ca²⁺) imaging.

**Results:**

hiPSC-derived SGN-like neurons exhibited morphological, molecular, electrophysiological, and functional characteristics of SGNs *in vivo*. Neurons acquired bipolar morphology and were wrapped by glial cells. Transcriptomic analysis revealed that SGN-like neurons were distinct from other neuronal lineages and showed similarity to type I and type II SGNs based on expression of synaptic and intrinsic excitability-related genes. Electrophysiological recordings revealed progressive hyperpolarization of resting membrane potential and emergence of overshooting action potentials, consistent with neuronal maturation. In co-culture systems, human SGN-like neurons formed functional synaptic connections with mouse cochlear hair cells and cochlear nucleus neurons, evidenced by Ca^2+^ transients and induction of the immediate early gene *c-Fos*.

**Conclusions:**

This study reports a robust and reproducible protocol for generating human SGN-like neurons from hiPSCs, providing a versatile platform for studying human auditory development, disease modeling, drug screening, and cell-based therapies for hearing restoration.

## 1 Background

The sensory hair cells that transduce sound within the mammalian inner ear are innervated by dendrites of spiral ganglion neurons (SGNs) [1], [2]. Axon bundles of SGNs project from the cochlea to the central nervous system as cranial nerve VIII to transmit auditory signals for higher processing [3], [4], [5]. Loss or damage of the SGNs due to diverse etiologies, including aging [6], [7], noise exposure [8], ototoxic drugs [9], infection [10], and hereditary defects [11], [12], is a leading cause of permanent hearing impairment in humans. SGN degeneration is often thought to occur secondarily due to the loss of hair cells that normally provide essential trophic support [13], [14]. However, several studies in humans have demonstrated that primary degeneration of SGNs can occur in the presence of morphologically intact or minimally damaged hair cells, particularly in cases of aging [15], exposure to certain ototoxic drugs [9], [16], viral infections [10], [17], certain rheumatologic conditions [10], and genetic mutations affecting neuronal survival [10], [18], [19] and synaptic transmission [12]. Notably, in age-related hearing loss, primary SGN degeneration accounts for an estimated 15%–30% of cases in humans [7], [15].

A fundamental obstacle in the study of human SGNs and the development of therapeutic strategies for their restoration is the lack of established and well-characterized *in vitro* models. It is not possible to non-destructively obtain human inner ear tissue biopsies due to the small size of the human cochlea, its complex three-dimensional (3D) anatomy, and encasement in dense bone. To overcome this challenge, there have been multiple attempts to create mouse primary SGN cell lines [20] or, more recently, to generate otic neurons from human stem cells [21], [22], [23], [24], [25], [26], [27], [28], [29]. To date, these methods have yielded cells of uncertain identity and function. In particular, the absence of SGN-specific markers, critical for defining human SGNs from other possible neuron types, has led to ambiguity regarding the identity of cells generated from pluripotent stem cells in prior protocols [21], [22], [23], [24], [25], [26]. Thus, there is an urgent need for further improvement in the method of SGN generation as well as a multi-factorial, quantitative understanding of the cells’ identity to establish their utility in otologic research.

Here, by mimicking the cell-to-cell signaling occurring during human inner ear development *in vivo*, we engineered an optimized protocol for the generation of functional human SGN-like neurons via otic neurosensory progenitor (ONP)-like cells derived from human induced pluripotent stem cells (hiPSCs). This study aimed to characterize their morphological, molecular, electrophysiological, and functional features to demonstrate their cellular identity and support their utility as effective *in vitro* models of human SGNs.

## 2 Methods

### 2.1 hiPSC culture

hiPSC lines SK8-A, generated in our laboratory [30], and UCSD112i-2-11 (UCSD), purchased from WiCell (Madison, WI, USA), were used before passage number 50. Cells were maintained on Matrigel human embryonic stem cell (hESC) qualified matrix (Cat# 354277, Corning, USA) in mTeSR1 (Cat# 85850, StemCell Technologies, Canada) or mTeSR plus medium (Cat# 100-0276, StemCell Technologies, Canada) supplemented with 1× penicillin-streptomycin (Cat# 15140122, Gibco, USA). Detailed culture conditions, reagents, and catalog numbers are provided in Additional file 1**: Methods**. Research involving hiPSC was reviewed by the Human Studies Committee of the Massachusetts General Brigham Institutional Review Board and approved on 24 May 2018 (14-148H) and 2 November 2018 (16-066H).

### 2.2 Differentiation of hiPSCs

For differentiation, we modified a previously published protocol [22] and optimized it for better differentiation into SGNs using chemically defined media (CDM). In our study, undifferentiated hiPSC lines SK8-A and UCSD were dissociated with TrypLE Select (Cat# 12563011, Gibco, USA) and seeded at 10,000 cells/cm^2^ (SK8-A) or 8000–10,000 cells/cm^2^ (UCSD) onto growth factor-reduced Matrigel (Cat# 356230, Corning, USA) in mTeSR. The concentration of Y-27632 (Cat# 1254, TOCRIS, UK), an inhibitor of Rho-associated protein kinase used to enhance stem cell survival in culture, was maintained at 10 μmol/L throughout D0–2. On D3, the medium was replaced with a CDM containing 10 ng/ml bone morphogenetic protein 4 (BMP4; Cat# 314-BP, R&D Systems, USA), 1 μmol/L SB-431542 (Cat# 1614, TOCRIS, UK), and 10 ng/ml basic fibroblast growth factor 2 (FGF2, Cat# 233-FB, R&D Systems, USA). The CDM contained a 50:50 mixture of DMEM/F12 (Cat# 11330032, Gibco, USA) and Neurobasal medium (Cat# 21103049, Gibco, USA), additionally supplemented with N2 [1% (v/v) final concentration, Cat# 17502048, Gibco, USA], B27 [2% (v/v) final concentration, Cat# 17504044, Gibco, USA], 2 mmol/L L-glutamine (Cat# 25030032, Gibco, USA), 0.1 mmol/L 2-mercaptoethanol (Cat# 21985023, Gibco, USA), and 50 ng/ml Normocin (Cat# ant-nr, InvivoGen, USA). On D6, the medium was changed to CDM supplemented with 100 nmol/L LDN-193189 (Cat# 04-0074-02, Stemgent, USA), 1 μmol/L SB-431542, 2 mmol/L IWP-2 (Cat# 3533, TOCIRS, UK), and 10 ng/ml FGF2.

On D11, TRA-1-60 (pluripotent stem cell marker)^-^/nerve growth factor receptor (NGFR)^+^ cells were collected by using magnetic sorting with anti-TRA-1-60 (Cat# 130-100-832, Miltenyi Biotec, Germany) and anti-NGFR (Cat# 130-097-127, Miltenyi Biotec, Germany) microbeads. A full version of the sorting protocol is available on Miltenyi Biotec’s website (https://www.miltenyibiotec.com/US-en/). After purification, the cells were seeded at 60,000 cells/cm^2^ on growth factor-reduced Matrigel-coated plates in CDM with 100 ng/ml Wnt3a (Cat# 5036-WN, R&D Systems, USA), 10 ng/ml FGF2, 50 ng/ml insulin-like growth factor-1 (IGF-1, Cat# 291-G1, R&D Systems, USA), and 10 μmol/L Y-27632. On D18, the medium was replaced with CDM containing 500 ng/ml sonic hedgehog (SHH, Cat# 1845-SH, R&D Systems, USA), 0.5 μmol/L retinoic acid (RA, Cat# 0695, TOCRIS, UK), 20 ng/ml epidermal growth factor (EGF, Cat# 236-EG, R&D Systems, USA), 10 ng/ml FGF2, and 50 ng/ml IGF-1. On D22, coverslips or culture plates were coated with 0.1 mg/ml poly-ornithine (Cat# P3655, Sigma, USA) diluted in 1× borate buffer (Cat# 28341, Thermo Scientific, USA) for >3 h at room temperature followed by 20 μg/ml laminin (Cat# 3400-010-02, R&D Systems, USA) in Hank’s Balanced Salt Solution (HBSS; Cat# 14025076, Gibco, USA) for 3 d at 4°C. During D3–25, the medium was changed every day. On D25, cells were treated with TrypLE Select and gently detached by cell scrapers. The cells were seeded at 500,000 cells/cm^2^ on poly-ornithine and laminin-coated coverslips or plates in Neurobasal medium supplemented with 1% N2, 2% B27, 1 mmol/L GlutaMAX (Cat# 35050061, Gibco, USA), 0.5 mmol/L dibutyryl-cyclic adenosine monophosphate (cAMP, Cat# SC-201567, Santa Cruz Biotechnology, USA), 10 ng/ml brain-derived neurotrophic factor (BDNF, Cat# 248-BDB, R&D Systems, USA), 10 ng/ml NT-3 (Cat# 267-N3, R&D Systems, USA), 10 ng/ml IGF-1, 50 ng/ml Normocin, and 10 ng/ml H1152 (Cat# 2414, TOCRIS, UK). The medium without H1152 was replaced every two days from D26 to D30. Half of the medium without H1152 was replaced every three days afterward. The compositions of the media are listed in the **Additional files 1 and 2**.

### 2.3 Human subjects

Human vestibular inner ear tissue was obtained during surgical labyrinthectomies and translabyrinthine resections of vestibular schwannomas (*n*=2). Tissue was used for immunohistological comparison with hiPSC-derived SGN-like neurons. Research involving human vestibular tissue obtained from patients was reviewed by the Massachusetts General Brigham Institutional Review Board and determined to be exempt from approval (2020P003329) on 2 December 2020.

### 2.4 Single-cell RNA sequencing (scRNA-seq)

SGN-like cells differentiated from SK8-A and UCSD hiPSCs were dissociated at multiple developmental time points (D25, D60, D90, and D120) and processed for scRNA-seq using the Chromium platform (10× Genomics). Libraries were prepared according to manufacturer protocols and sequenced to sufficient depth to ensure robust transcriptomic coverage. Vendor-specific protocols, quality control steps, and sequencing parameters are detailed in Additional file 1**: Methods**.

### 2.5 scRNA-seq data analysis

Raw sequencing data were processed using the 10× Genomics Cell Ranger 2.1.0 pipeline (http://support.10xgenomics.com/) and analyzed in Seurat. Low-quality cells and doublets were removed based on standard filtering criteria. Dimensionality reduction, clustering, and marker identification were performed using established workflows. Cross-species integration with published mouse datasets was conducted using Harmony to assess developmental correspondence. Detailed filtering thresholds, software versions, and analysis parameters are provided in Additional file 1**: Methods**.

### 2.6 Immunocytochemistry and imaging

Cells and co-culture samples were fixed, immunolabeled, and imaged using confocal microscopy. Standard blocking, antibody incubation, and imaging procedures were used. Z-stack imaging and 3D reconstruction were applied where appropriate to assess synaptic contacts. Antibody lists, concentrations, and imaging parameters are provided in **Additional files 1 and 3**.

### 2.7 Electrophysiological recordings

Whole-cell patch-clamp recordings were performed on hiPSC-derived SGN-like neurons at D88–95 or D120–234. Neuronal intrinsic properties, action potential (AP) firing patterns, and voltage-gated currents were assessed under current- and voltage-clamp configurations. Recording solutions, stimulation paradigms, and analysis criteria followed established protocols and are detailed in Additional file 1**: Methods**.

### 2.8 Animal models and co-culture systems

A total of 36 mice of either sex were used in this study, including 31 CBA/CaJ wild-type mice and 5 NOD/SCID wild-type mice. Of these, 35 mice were postnatal (P3–6), and 1 mouse was adult (8 weeks). Postnatal and adult mice were used for cochlear explant and cochlear nucleus (CN) co-culture experiments. Explants were positioned near hiPSC-derived SGN-like neurons to assess neurite outgrowth and synaptic connectivity. Cultures were maintained under controlled conditions and analyzed by immunofluorescence. Animal numbers, dissection procedures, and co-culture details are provided in Additional file 1**: Methods**. Mice were produced from Jackson Laboratory (Bar Harbor, ME, USA). All experimental procedures with mice were approved by the Institutional Animal Care and Use Committees of Massachusetts Eye and Ear Infirmary in Boston, MA, USA, and Stanford University in Stanford, CA, USA (approved protocol ID: 33998). All procedures with animals were conducted according to the National Academies of Sciences Guide for the Care and Use of Laboratory Animals (8th edition, NAS Press, USA).

### 2.9 Ca^2+^ imaging

Ca^2+^ imaging was used to assess synaptic activity in human SGN-like neurons co-cultured with mouse hair cells or CN neurons. Fluorescent Ca²⁺ indicators were applied, and activity was recorded before and after pharmacological blockade of glutamatergic transmission. Imaging parameters and quantitative analysis methods are detailed in Additional file 1**: Methods**.

### 2.10 Statistical analysis

Statistical analyses were performed using GraphPad Prism, with significance defined as *P*<0.05. Exact *P* values, sample sizes, and replicate information are reported in figures, tables, and Additional file 1**: Methods**.

## 3 Results

### 3.1 Generation of human ONP-like cells from hiPSCs

**Fig. 1:**
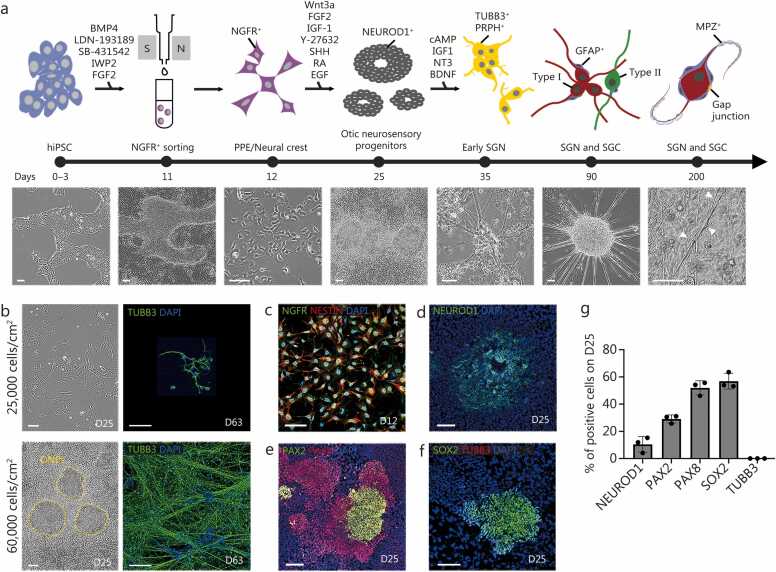
Stepwise differentiation of hiPSCs into human ONPs. **a** Overview and timeline of human SGN-like neuron differentiation protocol from hiPSCs and matching bright-field images. Scale bar=100 μm. **b** Comparison of cells at D25 and D63 following seeding at different cell densities (25,000 or 60,000 cells/cm^2^) on D11 after sorting with NGFR beads. Scale bar=100 μm. **c** Immunostaining for NGFR^+^NESTIN^+^ precursors of PPE and neural crest cells at D12. Scale bar=100 μm. **d**-**f** Immunostained D25 cells. Antibodies highlight neuroblast cells (NEUROD1^+^) and otic lineage cells (PAX2^+^, PAX8^+^, and SOX2^+^), but no expression of neuronal markers (TUBB3) at this stage. Scale bar=100 μm. **g** Bar graphs showing the average proportion (%) of positive cells on D25 cells stained with NEUROD1, PAX2, PAX8, SOX2, and TUBB3. Error bars represent SD. hiPSCs. Human induced pluripotent stem cells; ONPs. Otic neural progenitors; BMP4. Bone morphogenetic protein 4; LDN. LDN-193189; IWP2. Inhibitor of Wnt production-2; FGF2. Basic fibroblast growth factor 2; NGFR. Nerve growth factor receptor; Wnt3. Wnt family member 3; IGF-1. Insulin-like growth factor 1; SHH. Sonic hedgehog; RA. Retinoic acid; EGF. Epidermal growth factor; NEUROD1. Neurogenic differentiation 1; cAMP. Cyclic adenosine monophosphate; NT3. Neurotrophin 3; BDNF. Brain-derived neurotrophic factor; TUBB3. Tubulin beta 3 class III; PRPH. Peripheral neuronal marker peripherin; MPZ. Myelin protein zero; PPE. Pre-placodal ectoderm; SGC. Satellite glial cells; D. Day; SD. Standard deviation; GFAP. Glial fibrillary acidic protein; PAX2. Paired box gene 2; SOX2. SRY-box transcription factor 2; DAPI. 4′;6-diamidino-2-phenylindole.

Mammalian inner ear cells and their neighboring cells arise from two embryonic origins: the otic placode from the pre-placodal region [31] and cranial neural crest (CNC) cells from the neural tube [32], [33]. The otic placode gives rise to an otic vesicle, which is the origin of most cell types in the inner ear. The cells in the otic vesicle undergo proliferation and then differentiation, with higher expression of SHH in the ventral versus dorsal cochlear duct specifying the future cochlea and vestibule, respectively [34], [35]. CNC cells are a source of glial cells and periotic mesenchyme in the inner ear *in vivo* [36], [37]. With these developmental cues in mind, we generated pre-otic fate cells and their neighboring cells, including pre-placodal ectoderm and neural crest cells, from hiPSCs (SK8-A) (Fig. 1**a;** Additional file 2). On D11 of differentiation, we performed magnetic-activated cell sorting to isolate NGFR^+^ cells, which are known to be enriched for pre-placodal ectoderm and neural crest [38], [39] during human development [40]. As the survival and appropriate motility of these cells are highly sensitive to cell-cell interactions [41] and low density promotes non-neural ectoderm [42], we improved upon a previous protocol [22] by optimizing the cell density at 60,000 cells/cm^2^ on D11 (Additional file 4).

After cell sorting, we observed that low seeding density (25,000 cells/cm^2^) of precursors of pre-placodal ectoderm and neural crest on D11 negatively impacted development into SGN-like cells (Additional file 1**:** Fig. S1a). The cell numbers gradually decreased beginning around D25, with only <5 cells/cm^2^ surviving by D63 (Fig. 1**b**). Low seeding density also negatively affected neurite length. The longest length of neurons stained with tubulin beta 3 class III (TUBB3) and neurofilament (NEFL), from axon to dendrite, was <200 μm (Fig. 1**b;** Additional file 1**:** Fig. S1b). We subsequently determined that seeding 60,000 cells/cm^2^ of precursors of pre-placodal ectoderm and neural crest on D11 after sorting was the appropriate cell density (Additional file 1**:** Fig. S1c, **d**); 98.3% [with standard deviation (SD) 1.8%] of sorted cells were NGFR^+^ on D12 (Fig. 1**c**). To induce ONP-like cells and mimic essential *in vivo* signaling cues ultimately directing to an SGN fate, the pre-placodal ectoderm and neural crest cells were grown in medium containing SHH between D18–25 [22]. This protocol reproducibly generated a donut-shaped cluster of ONP-like cells [neurogenic differentiation 1 (NEUROD1)^+^] and otic progenitors [paired box 2 (PAX2)^+^, PAX8^+^, and SRY-box transcription factor 2 (SOX2)^+^] (Fig. 1**d**-**g**), which was replicated in another hiPSC cell line (UCSD) (Additional file 1**:** Fig. S1e-**h**).

### 3.2 Generation of neurons with human SGN-like properties from ONP-like cells

Under a cocktail of BDNF, neurotrophin-3 (NT3), and IGF-1, which are endogenous neurotrophins essential for the development and maintenance of SGNs [22], [43], neurites began to grow out from the neuronal body around D35. Between D35 and D90, the neurites transitioned from having complex branching patterns to a classic bipolar configuration as small branches disappeared (Additional file 1**:** Fig. S2), resembling the loss of branches *in vivo* as SGNs extend their neurites into the cochlea and central nervous system [44]. At D90, the expression of the glutamatergic neuronal marker vesicular glutamate transporter 1 (VGLUT1), which packages glutamate into synaptic vesicles, further suggested that these hiPSC-derived neurons have shared properties with SGNs (Additional file 1**:** Fig. S2d, **m**).

**Fig. 2:**
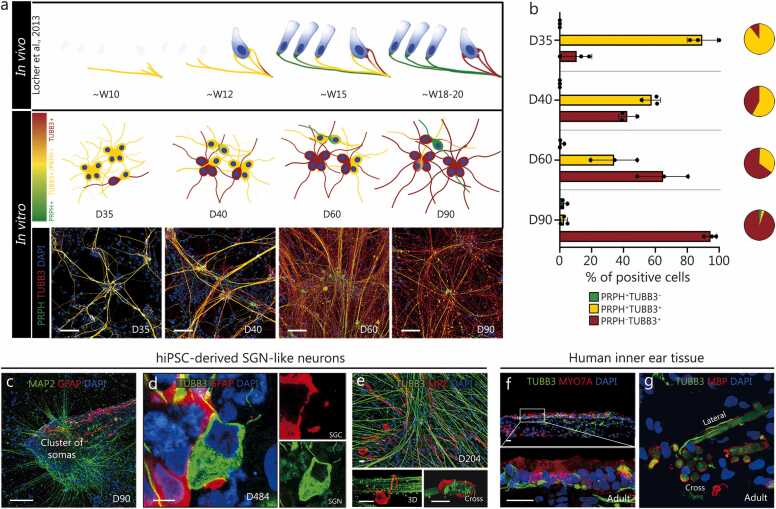
Differentiation of human SGN-like neurons *in vitro* mimics cochlear neuron development *in vivo*. **a** Comparison of human SGN development *in vivo* (as described by Locher et al. [49]) with corresponding stages of *in vitro* differentiation from hiPSCs. At W12 of *in vivo* development, the first inner hair cells appear and are contacted by multiple neurites co-expressing TUBB3 (red) and PRPH (green), with co-localization indicated in yellow. A comparable stage is observed around D40 of *in vitro* differentiation. By W20 *in vivo*, both inner and outer hair cells are present, and PRPH expression distinguishes type I (PRPH^-^) from type II (PRPH^+^) neurites, paralleling the differentiation state observed around D90 *in vitro*. Scale bar=100 μm. **b** Bar graphs and pie charts showing the average proportion (%) of TUBB3^+^ and/or PRPH^+^ somata in human SGN-like neurons at D35–90. **c** Overall view of the morphology of human SGN-like neurons, depicted by microtubule-associated protein 2 (MAP2) expression along with glial cell marker GFAP on D90. Scale bar=100 μm. **d** GFAP^+^ cell surrounding the cell body of a type I SGN-like neuron (TUBB3^+^) on D484. Scale bar=5 μm. **e** Expression of MPZ in cultures at D204. Lower panels show three-dimensional (3D) and cross-sectional views. Scale bar = 20 μm. **f**, **g** Surgical specimens of human vestibular end organs from the inner ear. MYO7A^+^ hair cells are innervated by TUBB3^+^ neurons (**f**), and TUBB3^+^ neurons are enveloped by MBP^+^ myelinating Schwann cells (**g**). Scale bar = 20 μm. SGN. Spiral ganglion neuron; hiPSCs. Human induced pluripotent stem cells; W. Week; TUBB3. Tubulin beta 3 class III; PRPH. Peripheral neuronal marker peripherin; D. Day; GFAP. Glial fibrillary acidic protein; MPZ. Myelin protein zero; DAPI. 4′;6-diamidino-2-phenylindole; MBP. Myelin basic protein; SGC. Satellite glial cells.

*In vivo*, mature SGNs consist of two main populations that are functionally distinct and can be identified by the absence (type I) or presence (type II) of the peripheral neuronal marker peripherin (PRPH) [45]. In the organ of Corti, the sensory epithelium of the cochlea, type I myelinated neurons innervate inner hair cells, whereas type II non-myelinating neurons innervate outer hair cells [1], [2], [46]. In our *in vitro* system, at D35, (89.4±9.5)% of early-stage human SGN-like neurons expressed both the pan-neuronal marker TUBB3 and PRPH, suggesting that specific type I and type II neurons are not differentiated at this stage in culture. However, PRPH expression was gradually lost between D35 and D90; at D90, (94.6±3.9)% of cells expressed only TUBB3 and not PRPH, fitting a molecular phenotype of type I SGNs (Fig. 2**a**, **b**). The small percentage of neurons that remained PRPH^+^ expressed additional markers of type II SGNs, such as GATA binding protein 3 (GATA3) or tyrosine hydroxylase, further supporting their molecular type II identity (Additional file 1**:** Fig. S2e, **f**). This differentiation process *in vitro* corresponds to both mouse [47], [48] and human [49] auditory neuron development in the cochlea. The protein expression pattern at approximately D35 was comparable to that seen at roughly around 10 weeks of gestation (W10), and approximately D90 was comparable to W18–20, which is when PRPH expression distinguishes type I (PRPH^-^) from type II (PRPH^+^) SGNs *in vivo* [49].

In the human cochlea, peripheral glial cells envelop or myelinate the SGNs and promote their survival, aid in synapse formation and pruning, and provide nutrient and metabolic support [50], [51]. We performed immunostaining to investigate whether the glial cells were engaged in a specific, organized structure with the human SGN-like neurons. From around D90, the glial cell marker glial fibrillary acidic protein (GFAP) was observed in clusters of SGN-like cell somas and in neurites (Fig. 2**c**). At higher magnification, the cell bodies of human SGN-like neurons were observed to be partially surrounded by satellite glia-like cells (Fig. 2**d**). In addition, 3D cross-section images showed that myelinating [myelin protein zero (MPZ)^+^] and non-myelinating (NGFR^+^) Schwann cells wrapped the axons of types I and II human SGN-like neurons, respectively (Fig. 2**e;** Additional file 1**:** Fig. S2g, **p**, **q**). The formation of myelinating fibers along human SGN-like neurons was comparable to Schwann cells’ ensheathment observed in human adult inner ear tissue [52] (Fig. 2**f**, **g**). Together, this evidence demonstrates that our culture system simulates the gradual morphological development of SGNs in the cochlea and their interaction with peripheral glial cells, as previously observed in humans [49]. Importantly, this differentiation process was phenocopied using the UCSD cell line (Additional file 1**:** Figs. S1, S2).

### 3.3 Lineage origins of the human SGN-like neurons

**Fig. 3:**
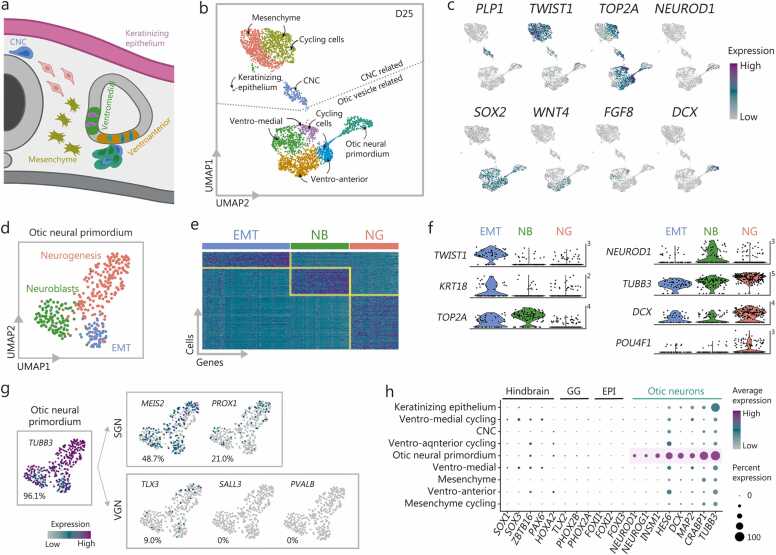
Origin of human SGN-like neurons revealed by scRNA-seq. **a** Schematic illustration of the developmental origins of auditory neurons in the human inner ear *in vivo*. **b** UMAP plots of SK8-A D25 cells, color-coded as in Fig. 3a. **c** Feature plots showing key gene markers for the classification of cell subtypes and determination of neuronal origins. **d** Re-clustering of isolated otic neural primordium cells from Fig. 3b, separating into three populations: EMT, delaminating neuroblasts, and neurogenesis. **e** Heatmap showing the top 100 differentially expressed genes across the three distinctive clusters: EMT, NB, and NG. The X and Y axes represent differentially expressed genes and single cells, respectively. **f** Violin plots displaying marker gene expression in a log-transformed scale among the three different clusters of otic neural primordium. The number in the upper right corner represents log-normalized gene expression (log1p normalized UMI counts) per cell. **g** Feature plot visualizing expression of SGN and VGN markers in the otic neural primordium cluster, based on scRNA-seq data from previous studies [59]. Numbers in the lower left corners indicate the percentage of cells expressing each marker. **h** Dot plots illustrating minimal expression of hindbrain, GG, and EPI markers, and high expression of otic neuroblast and neuronal markers. To support data sharing, visualization, and analysis, the processed scRNA-seq dataset for Fig. 3 is available on the gEAR portal (https://umgear.org/p?l=sk8aNeuron). SGN. Spiral ganglion neuron; scRNA-seq. Single-cell RNA sequencing; UMAP. Uniform manifold approximation and projection; EMT. Epithelial-mesenchymal transition; NB. Neuroblasts; NG. Neurogenesis; VGN. Vestibular ganglion neurons; GG. Geniculate ganglion; EPI. Epibranchial placode; PLP1. Proteolipid protein 1; TWIST1. Twist family bHLH transcription factor 1; TOP2A. DNA topoisomerase II alpha; MEUROD1. Neuronal differentiation 1; SOX2. SRY-box transcription factor 2; WNT4. Wnt family member 4; FGF8. Fibroblast growth factor 8; DCX. Doublecortin; MEIS2. Meis homeobox 2; PROX1. Prospero homeobox 1; TLX3. T cell leukemia homeobox 3; SALL3. Spalt like transcription factor 3; PVALB. Parvalbumin; CNC. Cranial neural crest; ZBTB16. Zinc finger and BTB domain containing 16; PAX6. Paired box 6; HOXA2. Homeobox A2; FOXI1. Forkhead box I1; PHOX2A. Paired mesoderm homeobox protein 2A; FOXI2. Forkhead box I2; NEUROG1. Neurogenin 1; INSM1. Insulinoma-associated 1; HE56. Hes family BHLH transcription factor 6; MAP2. Microtubule-associated protein 2; TUBB3. Tubulin beta 3 class III.

To understand the molecular diversity of cell lineages arising in developing auditory neurons (Fig. 3**a**), we performed scRNA-seq at D25. Downstream analyses were conducted after initial quality control and doublet removal (Additional file 1**:** Fig. S3a**-g**). D25 clustering (3896 cells), visualized via uniform manifold approximation and projection (UMAP), suggested two transcriptionally distinct groups of cells originating from the proteolipid protein 1 (*PLP1*)^*+*^ CNC (43.7%) and *SOX2*^*+*^ otic vesicle (55.8%) (Fig. 3**b;** Additional file 5).

The composition of the cell types translated to a differentiation efficiency rate of 99.5% inner ear-related cells and only 0.5% keratinizing epithelium. The CNC-originated group comprised three clusters: CNC cells [*PLP1*^+^ Forkhead box D3 (*FOXD3*)^+^, 6.0%], mesenchyme [Twist-related protein 1 (*TWIST1*)^+^ *Lumican* (*LUM*)^+^ *TGFBI*^+^ Decorin (*DCN*)^+^, 20.3%], and mesenchyme-cycling [*TWIST1*^+^*LUM*^+^*TGFBI*^+^*DCN*^+^ DNA topoisomerase II alpha (*TOP2A*)^+^, 17.4%] (Fig. 3**c**), similar to prior reports in mouse [53]. Periotic mesenchymal cells are the most numerous cell types within the cochlea, influencing SGN peripheral axon formation, organization, and innervation *in vivo*; gene mutations in these cells cause hearing loss [11]. Accordingly, more than one-third (37.7%) of the mesenchymal population in this culture is expected to support the generation of SGN-like cells (Fig. 3**b**). No cell clusters expressed early mesodermal or endodermal lineage markers such as T-box transcription factor T (*TBXT*) or *SOX17*, suggesting that the mesenchyme cells in the culture arose from a CNC rather than a mesodermal lineage.

The otic vesicle-originating group diverged into otic vesicle-like cells corresponding to ventro-anterior [Wnt family member 4 (*WNT4*)^+^ Hes family bHLH transcription factor 5 (*HES5*)^+^, 27.7%] and ventro-medial [Fibroblast growth factor 8 (*FGF8*)^+^ *PAX2*^*+*^, 18.9%] fates *in vivo* (Fig. 3**b, c;** Additional file 5) [54]. The ventro-anterior cells were closely aligned with cochleovestibular ganglia (CVG), resembling *in vivo* neuroblasts (the origin of SGNs and vestibular ganglion neurons), delaminating from the otic vesicle through epithelial-mesenchymal transition (EMT) and differentiating into neurons, as previously described [55]. No cell clusters expressed markers involved in suppressing CVG formation, such as TBX1, in the dorsal domain of the otic vesicle [56]. As *in vivo*, the cluster of otic neural primordium consisted of EMT cells [*TWIST1*^+^ Keratin 18 (*KRT18*)^+^ Vimentin (*VIM*)^+^], delaminating neuroblasts (*NEUROD1*^+^*HES6*^+^), and cells undergoing neurogenesis [*TUBB3*^+^ Doublecortin (*DCX)*^+^ POU class 4 homeobox 1 (*POU4F1*)^+^] (Fig. 3**d**-**f**). Similar to observations from mouse E10.5 neuroblasts [57], the broad expression of neuronal marker *TUBB3* was evident in otic neural primordium, particularly in the neurogenesis cluster; DCX, a late neuroblast- and young neuron-marker [58], was also most abundantly expressed in the neurogenesis cluster (Fig. 3**f**). Additionally, cells involved in EMT and neuroblast phases exhibited high expression of proliferative markers such as *TOP2A* (Fig. 3**f**). However, as the neurogenesis process progressed, these cells lost their proliferative capacity, differentiating into neurons. Within the otic neural primordium cluster, a larger proportion of these neurons expressed genes suggesting SGN-like identity, such as meis homeobox 2 (*MEIS2*, 48.7%) and prospero homeobox protein 1 (*PROX1*, 21.0%), compared to those associated with early vestibular ganglion neurons such as T cell leukemia homeobox 3 (*TLX3*, 9.0%), spalt like transcription factor 3 (*SALL3*, 0%), and parvalbumin (*PVALB*, 0%) [59] (Fig. 3**g**). Additionally, 41.7% of the cells expressed *INSM1* (Additional file 5), which is reported to be expressed in both otic neural progenitors and early SGNs [60]. The low expression of genes specific to hindbrain neurons, geniculate ganglion of the facial nerve adjacent to the inner ear, and epibranchial placode (Fig. 3**h**) reinforces the specificity of the auditory neuron differentiation. To ensure experimental robustness, we repeated the scRNA-seq experiments with another cell line (UCSD) on D25, yielding consistent results (Additional file 1**:** Fig. S3h**-j**).

### 3.4 Diversity of human SGN-like neurons

**Fig. 4:**
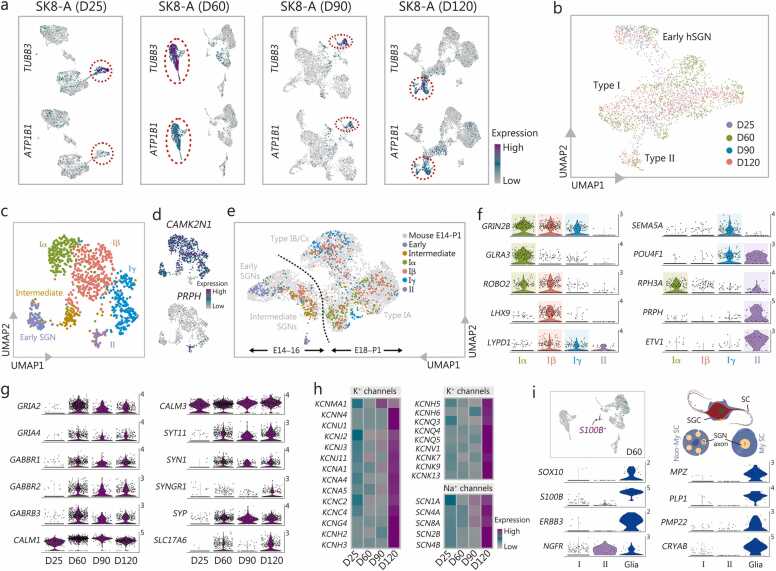
Diversity of human SGN-like neurons revealed by scRNA-seq. **a** Feature plot visualizing expression of neuronal markers *TUBB3* and *ATP1B1* in D25, D60, D90, and D120 scRNA-seq data. Neuronal clusters indicated by red dotted circles were isolated for a downstream analysis. **b** UMAP visualization of neurons isolated from each time point. Purple dots represent D25 neurons, which did not clearly segregate into type II populations. **c** UMAP plots revealing 6 distinctive clusters of D60 human SGN-like neurons. **d** Identification of type I and II human SGN-like neuron clusters based on *CAMK2N1* and *PRPH* expression, respectively. **e** Comparison of D60 human SGN-like neuron subtypes with developmental stages of SGNs in E14–P1 mice [48] projected onto UMAP dimensions. **f** Violin plots displaying log-transformed expression levels of marker genes across 4 distinct human SGN-like neuron populations. **g** Expression profiles of selected genes related to glutamate and GABA receptors, synaptic vesicles, neurotransmitter transporters, and Ca^2+^ binding proteins among D25, D60, D90, and D120 human SGN-like neurons. **h** Differential expression patterns of K^+^ and Na^+^ channels among D25, D60, D90, and D120 human SGN-like neurons. **i** UMAP plots depicting *S100B*^+^ peripheral glial cell cluster in the D60 sample and expression of their gene markers. The number in the upper right corner represents log-normalized gene expression (log1p normalized UMI counts) per cell (**f, g, i**). To support data sharing, visualization, and analysis, the processed scRNA-seq dataset for Fig. 4 is available on the gEAR portal (https://umgear.org/p?l=sk8aNeuron). SGN. Spiral ganglion neuron; scRNA-seq. Single-cell RNA sequencing; D day; UMAP. Uniform manifold approximation and projection; SGC. Satellite glial cell; Non-my SC. Non-myelinating Schwann cells; My SC. Myelinating Schwann cells; PRPH. Peripheral neuronal marker peripherin; Ca^2+^. Calcium ion; TUBB3. Tubulin beta 3 class III; ATP1B1. Sodium/potassium-transporting ATPase subunit beta-1; CAMK2N1. Calcium/calmodulin dependent protein kinase II inhibitor 1; E. Embryonic day; P. Postnatal day; GABA. Gamma-aminobutyric acid; GRIN2B. Glutamate ionotropic receptor NMDA Type subunit 2B; GLRA3. Glycine receptor alpha 3; ROBO2. Roundabout guidance receptor 2; LHX9. LIM Homeobox 9; LYPD1. LY6/PLAUR Domain containing protein 1; SEMA5A. Semaphorin 5A; POU4F1. POU class 4 homeobox 1; RPH3A. Rabphilin 3A; PRPH. Peripherin; ETV1. ETS variant transcription factor 1; GRIA2. Glutamate ionotropic receptor AMPA type subunit 2; GRIA4. Glutamate ionotropic receptor AMPA type subunit 4; GABBR1. Gamma-aminobutyric acid type B receptor subunit 1; GABBR2. Gamma-aminobutyric acid type B receptor subunit 2; GABRB3. Gamma-aminobutyric acid type A receptor subunit beta 3; CALM1. Calmodulin 1; CALM3. Calmodulin 3; SYT11. Synaptotagmin 11; SYN1. Synapsin I; SYNGR1. Synaptogyrin 1; SYP. Synaptophysin; SLC17A6. Solute carrier family 17 member 6; KCNMA1. Potassium calcium-activated channel subfamily M alpha 1; KCNN4. Potassium calcium-activated channel subfamily N member 4; KCNU1. Potassium Calcium-Activated Channel Subfamily U Member 1; KCNJ2. Potassium inwardly rectifying channel subfamily J member 2; KCNJ3. Potassium inwardly rectifying channel subfamily J member 3; KCNJ11. Potassium inwardly rectifying channel subfamily J member 11; KCNA1. Potassium voltage-gated channel subfamily A member 1; KCNA4. Potassium voltage-gated channel subfamily A member 4; KCNA5. Potassium voltage-gated channel subfamily A member 5; KCNC2. Potassium voltage-gated channel subfamily C member 2; KCNC4. Potassium voltage-gated channel subfamily C member 4; KCNG4. Potassium voltage-gated channel modifier subfamily G member 4; KCNH2. Potassium voltage-gated channel subfamily H member 2; KCNH3. Potassium voltage-gated channel subfamily H member 3; KCNH5. Potassium voltage-gated channel subfamily H member 5; KCNH6. Potassium voltage-gated channel subfamily H member 6; KCNQ3. Potassium voltage-gated channel subfamily Q member 3; KCNQ4. Potassium voltage-gated channel subfamily Q member 4; KCNQ5. Potassium voltage-gated channel subfamily Q member 5; KCNV1. Potassium voltage-gated channel modifier subfamily V member 1; KCNK7. Potassium two pore domain channel subfamily K member 7; KCNK9. Potassium two pore domain channel subfamily K member 9; KCNK13. Potassium two pore domain channel subfamily K member 13; SCN1A. Sodium voltage-gated channel alpha subunit 1; SCN4A. Sodium voltage-gated channel alpha subunit 4; SCN8A. Sodium voltage-gated channel alpha subunit 8; SCN2B. Sodium voltage-gated channel beta subunit 2; SCN4B. Sodium voltage-gated channel beta subunit 4; SOX10. SRY-box transcription factor 10; S100B. S100 Calcium binding protein B; ERBB3. Erb-b2 receptor tyrosine kinase 3; NGFR. Nerve growth factor receptor; MPZ. Myelin protein zero; PLP1. Proteolipid protein 1; PMP22. Peripheral myelin protein 22; CRYAB. Crystallin alpha B.

To map a transcriptional trajectory of the development of the human SGN-like neurons, scRNA-seq was conducted at three additional differentiation time points: D60, D90, and D120. The neuron clusters were isolated based on the expression of a range of pan-neuronal markers such as *TUBB3* and sodium/potassium-transporting ATPase subunit beta-1 (*ATP1B1*) (Fig. 4**a**). Although the UMAP visualization of the entire dataset suggests maturity progression from D25 to D60, D90, and D120, with the absence of a type II cluster at D25, we did not observe distinct, segregating clusters during D60–120 (Fig. 4**b**). This implies a similar gene expression pattern among D60–120 stages, albeit with some changes insufficient to delineate new distinct developmental clusters.

At D60, the efficiency rate of inner ear-related cell differentiation remained high (98.4%) in culture. Examining human SGN-like neuron heterogeneity, we visualized isolated *TUBB3*^+^ clusters (26.1%) via UMAP, revealing 6 distinct neuronal clusters, including early and intermediate human SGN-like neurons (Fig. 4**c**). Presumed types I and II human SGN-like neurons fell into distinct groups enriched for genes such as calcium/calmodulin-dependent protein kinase II inhibitor 1 (*CAMK2N1*) and *PRPH* (Fig. 4**d**). Overall, types I and II human SGN-like neurons composed 94.7% and 5.3% of the cell population, respectively, similar to histological estimates of their proportions *in vivo* [61].

To determine whether the human SGN-like neurons are undergoing differentiation or if specific subtypes are emerging, we conducted a correlation analysis between our D60 human SGN-like neurons and previously published data from E14–P1 mouse SGNs [48]. Early and intermediate human SGN-like neurons closely aligned with E14–16, while type I neurons overlapped with E18–P1, a stage at which all SGN subtypes are transcriptionally identifiable (Fig. 4**e;** Additional file 1**:** Fig. S4a). Indeed, the potential type I neurons could be further separated based on unique and combinatorial molecular profiles into clusters α [glycine receptor alpha 3 (*GLRA3*)^+^, roundabout guidance receptor 2 (*ROBO2*)^+^, and rabphilin 3 A (*RPH3A*^+^)], β [*ROBO2*^+^*,* LIM homeobox 9 (*LHX9*)^+^, and LY6/PLAUR domain containing 1 (*LYPD1*)^*+*^], and γ [*LYPD1*^*+*^*,* semaphorin 5 A (*SEMA5A*)^*+*^, and *POU4F1*^+^] (Fig. 4**f;** Additional file 6), although it is premature to conclude whether the clusters represent various differentiation stages or are comparable to mature SGN subtypes. In addition, each sub-cluster had differential expression of genes related to neuronal transmission, including synaptic vesicles, neurotransmitter transporters, Ca^2+^ binding proteins, and neurotransmitter receptors; and the difference was usually more prominent between types I and II (Additional file 1**:** Fig. S4b, **c**).

Regarding afferent cochlear neurotransmission, the primary neurotransmitter released by hair cells is glutamate [62], [63]. Our findings revealed the expression of various glutamatergic receptor subunits known to be expressed in SGNs (Fig. 4**g;** Additional file 1**:** Fig. S4e). Glutamate ionotropic receptor AMPA type subunit 2 (GRIA2) and subunit 4 (GRIA4) are the two main α-amino-3-hydroxy-5-methyl-4-isoxazolepropionic acid (AMPA) receptor subunits found in SGNs [64]. GRIA2 makes AMPA receptors Ca^2+^-impermeable and is postulated to be protective from glutamatergic excitotoxicity and hearing loss later in life [65]. Expression of *GRIA3*, although lower than that of *GRIA2* and *GRIA4* (Additional file 1**:** Fig. S4e), is important for regulating the AMPA receptor subunit stoichiometry in ribbon synapses, modulating ribbon morphology, and minimizing GluA2-lacking AMPA receptors that flux Ca^2+^ [65]. Glutamate ionotropic receptor NMDA type subunit 2B (GRIN2B) is an early developmental N-methyl-D-aspartate (NMDA) receptor subunit responsible for neuronal survival and glutamatergic transmission [66]; its expression in SGNs has been demonstrated previously in rats [64]. In addition to glutamatergic receptors, we report high expression of gamma-aminobutyric acid (GABA)-B receptors in both the type I and type II human SGN-like neurons (Fig. 4**g**; Additional file 1**:** Fig. S4f), as reported previously in rodents [67]. GABA-B receptors are found in SGN afferents under inner and outer hair cells, and these receptors retrogradely modulate the outer hair cells’ amplifier function in type II SGNs [67]. The expression of synaptic receptors remained relatively constant during D60–120 (Additional file 1**:** Fig. S4d**-f**).

Expression of synaptic vesicle-associated protein release machinery (e.g., *SYT11*, *SYN1*, and *SYNGR1*), neurotransmitter transporters (e.g., *SLC17A6*), and Ca^2+^ binding proteins (e.g., *CALM1* and *CALM3*) were also found in the human SGN-like neurons (Fig. 4**g**). Together, the transcriptomic data indicate that the human SGN-like neurons acquire a “kit” of synaptic receptors and vesicle release molecules that enable human SGN-like neurons to receive glutamatergic excitatory transmission, modulate their synaptic partners via GABA-B metabotropic receptors, and release glutamate themselves.

The maturity of the human SGN-like neurons is also reflected in the set of ion channel subunits they express [68], [69]. Ion channel composition determines neuronal intrinsic excitability and spiking properties, and modulates integration of synaptic inputs [68]. The human SGN-like neurons strongly up-regulated various potassium (K^+^) and sodium (Na^+^) channel subunits (Fig. 4**h**) around D120, much later than the observed expression of synaptic receptors and proteins, suggesting that neuronal intrinsic excitability matures *in vitro* after D120.

In addition to the human SGN-like neurons, there was a glia cluster (1.2% of cells) expressing pan-Schwann cell markers [S100 calcium binding protein B (*S100B*) and Erb-B2 receptor tyrosine kinase 3 (*ERBB3*)], a key Schwann cell lineage transcription factor (*SOX10*), myelin proteins (*PLP1* and *MPZ*), and other regulators of myelination [crystallin alpha B (*CRYAB*)] (Fig. 4**i**). A subset of these cells (31.9%) also expressed *NGFR* (Fig. 4**i**), a marker of type II SGNs, non-myelinating Schwann cells, and satellite glial cells [70].

Taken together, these findings suggest that the human SGN-like neurons generated via our method are comparable to type I and type II *in vivo* based on their transcriptomic signatures of synaptic and intrinsic excitability-mediating genes, alongside a distinct population of surrounding glial cells.

### 3.5 Intrinsic excitability of human SGN-like neurons: electrophysiological characterization

**Fig. 5:**
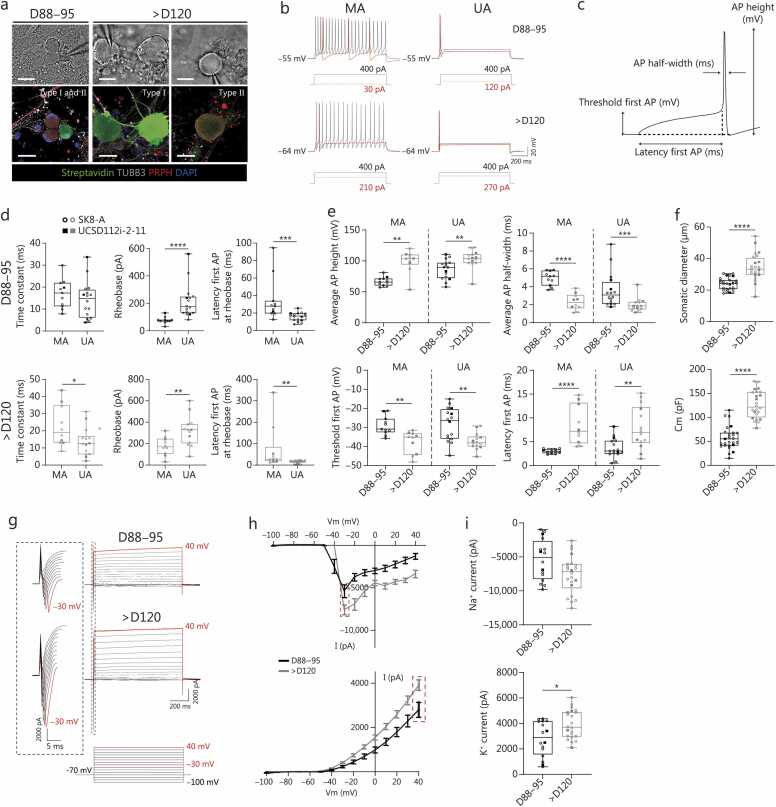
Electrophysiological characterization of human SGN-like neurons. **a** IR-DIC images during patch-clamping (top) and corresponding confocal images (bottom) identifying type I and type II human SGN-like neurons recorded at D88–95 and >D120 *in vitro*. Scale bar=25 μm. **b** Representative traces of current-clamp recordings in MA and UA type I human SGN-like neurons at D88–95 and >D120. Red traces represent the current step corresponding to the rheobase. **c** Schematic illustrating AP parameters analyzed. **d** Comparison of membrane time constant, rheobase, and latency to the first observed AP at rheobase between MA- and UA-type I human SGN-like neurons. Sample sizes: D88–95, MA (*n*=11), UA (*n*=16); >D120, MA (*n*=10), and UA (*n*=14). The circles and squares i*n*dicate SK8-A and UCSD cell lines, respectively, across all patch clamping datasets. **e** AP properties of type I human SGN-like neurons at the maximum injected current step. Sample sizes: D88–95, MA (*n*=11), UA (*n*=16); >D120, MA (*n*=10), UA (*n*=14). **f** Cell body diameter (top) and membra*n*e capacitance (Cm; bottom) of type I human SGN-like neurons. Sample sizes: D88–95 (*n*=27), >D120 (*n*=24). **g** Representative traces of voltage-clamp recordi*n*gs in type I human SGN-like neurons at D88–95 (top) and >D120 (bottom); red traces indicate maximum responses. **h** Voltage-current plots for putative Na^+^- (top) and K^+^-mediated currents (bottom); red dashed squares indicate maximum^-^evoked amplitudes. **i** Comparison of maximum-evoked amplitudes of putative Na^+^- (top) and K^+^-mediated currents (bottom) in type I human SGN-like neurons. Sample sizes: D88–95 (*n*=16), >D120 (*n*=22). ^⁎^*P*<0.05, ^⁎⁎^*P*<0.01, ^⁎⁎⁎^*P*<0.001, ^⁎⁎⁎⁎^*P*<0.0001. Error bars refer to the standard error of the mean (standard error of mean) in all panels. AP. Action potential; SGN. Spiral ganglion neuron; IR-DIC. Representative infrared differential interference contrast; MA. Multi-spike accommodating neurons; UA. Unitary-spike accommodating neurons; Vm. Membrane potential.

Whole-cell patch-clamp was used to determine whether the human SGN-like neurons can elicit APs and evaluate other electrophysiological properties important for overall excitability. Recordings were performed on human SGN-like neurons generated from SK8-A and UCSD hiPSC lines at two time points, D88–95 and D120–235 (>D120), to compare parameters before and after up-regulation of K^+^- and Na^+^-channel subunits (Fig. 4**h**). During recordings, human SGN-like neurons were filled with biocytin for post-hoc classification of either type I or type II by staining for PRPH (Fig. 5**a**). Type II human SGN-like neurons constituted approximately 10% of the recorded cells (4/31 at D88–95 and 2/26 at >D120), as reported *in vivo* [69]; therefore, we focused on characterizing the more prevalent type I neurons (approximately 90% of recorded cells).

First of all, we tested for spiking response patterns in current-clamp mode. Two distinct neuronal groups were revealed based on their responses to step current injections: multi-spike accommodating neurons [MA; *n*=21 (41%)] and unitary-spike accommodating neurons [UA; *n*=30 (59%)] (Fig. 5**b**). UA neurons did not fire more than two APs in response to current steps across a range of stimuli intensities, while MA neurons fired multiple APs and increased their firing frequency with incremental current amplitudes (Additional file 1**:** Fig. S5a). These results indicate that, like developing SGNs from other mammalian species, type I human SGN-like neurons exhibit two main subclasses based on their firing properties, likely coinciding with the expression pattern of different K^+^- and H-type currents [68], [71], [72], [73]. MA and UA type I human SGN-like neurons differed in their membrane time constant, rheobase (the minimum current intensity necessary to evoke an AP), and latency to the first evoked AP (time constant statistically different only at >D120) (Fig. 5**c**, **d**). These findings are consistent with prior reports in rodent SGNs [71]. Notably, the rheobase values increased by almost 2-fold between D88–95 and >D120 (Fig. 5**d**), along with hyperpolarized resting membrane potential (Additional file 1**:** Fig. S5d**;** Additional file 7), consistent with the upregulation of K^+^ channels (i.e., KCNQs) responsible for maintaining negative resting membrane potential [74] (Fig. 4**h**).

Given the increase in the expression of Na^+^ and K^+^ channel subunits responsible for fast rise [e.g., sodium voltage gated channel alpha subunit 1 (*SCN1A*) coding for Nav1.1] and rapid repolarization [e.g., potassium voltage gated channel subfamily A member (*KCNA*)*1*–*5* for Kv1–5 and potassium voltage gated channel modifier subfamily G member 4 (*KCNG4*) for Kv6.4.] of APs, and enabling high frequency firing [potassium voltage gated channel subfamily C member 2 (*KCNC2*) and 4; Kv3.2 and 3.4 [75]] in the human SGN-like neurons between D90 and D120 (Fig. 4**h**), we compared changes in several AP parameters between these timepoints to see if the direction of changes is as predicted by the transcriptome (Fig. 5**c, e;** Additional file 1**:** Fig. S5b**, c;** Additional file 7). Indeed, as Na^+^ channel subunit expression [e.g., sodium voltage-gated channel alpha subunit 1 (*SCNA1A*)] increased from D90 to D120, firing threshold lowered and AP amplitude almost doubled for both MA and UA, resulting in an overshooting AP characteristic of neurons *in vivo*. AP half-width decreased along with an increase in K^+^ channel abundance (Kv1 and Kv3 channels; KCNAs and KCNCs) and their lower activation threshold (KCNG4 subunit) [71], [76] (Fig. 5**e**). In addition to the changing active properties due to ion channel composition, passive properties also changed; between D88–95 and >D120, there was an increase in somatic diameter, and consequently, membrane capacitance (Cm) was increased (Fig. 5**f;** Additional file 7). An increase in Cm likely contributed to the longer latency to first AP (Fig. 5**e**).

To directly evaluate changes in ion channel currents, we performed whole-cell recordings of type I human SGN-like neurons in voltage-clamp configuration. We observed fast inward currents that peaked at holding voltages of –30 mV and long-lasting steady outward currents (Fig. 5**g, h**), which is consistent with the reported current-voltage (I–V) profiles for inward Na^+^- and Ca^2+^-current and outward K^+^-mediated currents in mouse and rat SGNs [71], [77]. The amplitudes of putative Na^+^- and K^+^-mediated currents were larger in >D120 as compared to D88–95 human SGN-like neurons (Fig. 5**g-i;** Additional file 7), in accordance with the increase in mRNA levels for these channels at these two differentiation time points (Fig. 4**h**). Type I human SGN-like neurons also exhibited a voltage sag in response to large hyperpolarizing current steps in current-clamp configuration, indicating the presence of the mixed conductance Na^+^/K^+^-mediated I_h_ currents as previously reported [78]. Consistent with the presence of the voltage sag, increasing expression of hyperpolarization-activated cyclic nucleotide-gated potassium channel (*HCN*)*1*–*3* subunits that build HCN channels was detected in increasing numbers of human SGN-like neurons from D60–120 (Additional file 1**:** Fig. S5e), similar to developing mouse ear [79]. Despite the increase in HCN expression, voltage sag significantly decreased between D88–95 and >D120 (*P*<0.0001), likely due to the increase in leak K^+^ channels, like potassium two pore domain channel subfamily K member 9 (*KCNK9*) (Fig. 4**h**), contributing to shunting of currents at membrane potentials around rest [80] (Additional file 1**:** Fig. S5f-**h;** Additional file 7).

Together, the results indicate that the active electrophysiological properties of the human SGN-like neurons coincide with the developmental increase in mRNA expression of ion channel subunits responsible for the reliable generation and propagation of APs (Nav1.1 and Kv1s), enabling and modulating high-frequency firing properties of critical importance for encoding auditory inputs [Kv1s, Kv3s, and human Ether-à-go-go-related gene (hERGs)] [71], [81], and fine-tuning of the neurons’ excitability (HCNs and leak channels). These changes, along with growth that affects passive properties of neurons, point to a maturation sequence for the human SGN-like neurons that results in functional profiles similar to those described for type I SGNs *ex vivo* [71], [72], [73].

### 3.6 Human SGN-like neurons form functional synapses with hair cells

**Fig. 6:**
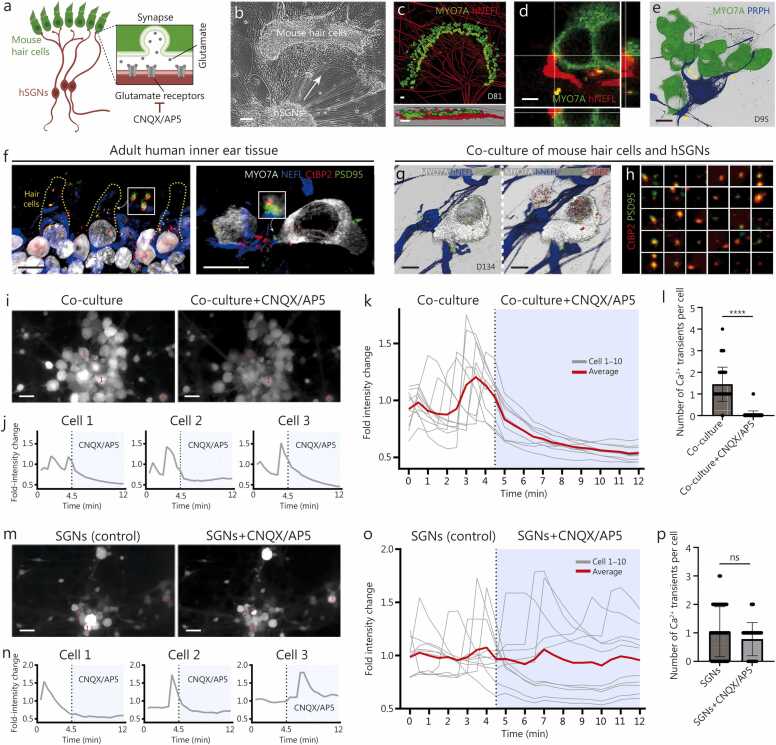
Human SGN-like neurons form functional synaptic connections with mouse hair cells. **a** A schematic illustrating the co-culture setup of human SGN-like neurons with mouse cochlear hair cells. **b** Bright-field images of denervated mouse hair cells co-cultured with human SGN-like neurons, placed at least 500 μm apart. Scale bar=100 μm. **c** In co-cultures, growth of D81 human SGN-like neurons (human-specific NEFL^+^) toward MYO7A^+^ hair cells (top) and side view of three-dimensional (3D) co-culture images (bottom). Scale bars=20 μm. **d** Higher magnification view of the contact site (x-y plane) along with orthogonal (x-z, y-z) projections, confirming tight proximity between hair cells and human SGN-like neurons. Scale bar=5 μm. **e** Multiple connections between PRPH^+^ type II D95 human SGN-like neurons and MYO7A^+^ mouse hair cells. Scale bar=10 μm. **f** Expression of presynaptic CtBP2^+^ and postsynaptic GLUR2^+^/PSD95^+^ proteins in surgical specimens of vestibular end organs from the human inner ear. Scale bar=10 μm. **g** Localization of CtBP2 and PSD95 synaptic puncta in co-cultures, along with MYO7A^+^ hair cells and hNEF^+^ human SGN-like neuron dendrites. Scale bar=5 μm. **h** Examples of paired synaptic (CtBP2 and PSD95) immunopuncta in co-culture of mouse hair cells and human SGN-like neurons, marking putative synapses (yellow overlap). **i** Fluorescent Cal520 Ca^2+^ signals from D193 human SGN-like neurons co-cultured with P5 mouse hair cells. Left: maximum intensity projection over 4.5 min. Right: maximum intensity projection over 7.5 min in the presence of CNQX and AP5 (AMPA/NMDA receptor blockers). Scale bar=100 μm. The numerals 1, 2, and 3 denote Cell 1, Cell 2, and Cell 3 used for analysis, respectively. **j** Representative Ca^2+^ transients from three human SGN-like neurons (**i**), showing loss of Ca^2+^ signal with CNQX/AP5 treatment. Ca^2+^ signals were normalized to the mean baseline intensity. **k** Representative experiment showing average relative change in Ca^2+^ fluorescence intensity (red line) from 10 human SGN-like neurons (D193; gray lines) co-cultured with P5 mouse hair cells. Ca^2+^ signal is abolished by CNQX and AP5. **l** Quantification of the number of Ca^2+^ transients in the co-culture before and after CNQX/AP5. Data from three preparations at D74, D80, D108, D193, and D264, for a total of *n*=60 neurons. **m-o** Parallel experiments in human SGN-like neurons solo culture at D193. CNQX and AP5 treatment does not abolish Ca^2+^ activity. The numerals 1, 2, and 3 in panel m denote Cell 1, Cell 2, and Cell 3 used for analysis, respectively. **p** Quantification of Ca^2+^ transients in human SGN-like neurons in solo culture. Data from three preparations at D74, D80, D108, D193, and D264, *n*=50 human SGN-like neurons. ^⁎⁎⁎⁎^*P*<0.0001 (Mann-Whitney test), ns not significant. SGN. Spiral ganglion neuron; IR-DIC. Representative infrared differential interference contrast; MA. Multi-spike accommodating neurons; UA. Unitary-spike accommodating neurons; D. Day; Ca^2+^. calcium ion; NEFL. Neurofilament; PRPH. Peripheral neuronal marker peripherin; P. Postnatal day; AMPA. Alpha-amino-3-hydroxy-5-methyl-4-isoxazolepropionic acid; NMDA. N-methyl-D-aspartate.

In mammals, SGNs are the bridge between the detection of physical sound by hair cells and the perception of that sound by the brain [1], [2]. Therefore, to validate the functionality of our human SGN-like neurons, we first examined their potential to form synaptic connections with hair cells, their only presynaptic partner *in vivo*. To achieve this, we used a mouse cochlear hair cell explant co-culture system (Fig. 6**a**) [23], [24], [26]. Mouse hair cell cochlear explants could potentially contain mouse SGNs. Therefore, before proceeding with the functional experiment, we confirmed the absence of mouse SGNs in our co-cultures by test-running explants from Thy1-GFP BL6 transgenic mice with GFP^+^ SGNs. Explants cultured for 14 d displayed no GFP-positive neurons, indicating that endogenous mouse SGNs did not survive in these conditions (Additional file 1**:** Fig. S6a). Therefore, any neuronal activity observed in co-cultures was exclusively due to the human SGN-like neurons.

For the co-culture experiment, CBA/CaJ mouse cochlear explants were dissected from the auditory neurons’ peripheral processes, thereby denervating the explant and leaving hair cells. After 14 d of solo culture of the denervated explant, mouse hair cells retained presynaptic ribbons, while the post-synaptic side was largely gone (Additional file 1**:** Fig. S6b), as previously reported [82]. Next, cells derived from our protocol (D81–134, which included SGN-like neurons as well as some mesenchyme and glial cells) were introduced to the denervated mouse hair cells culture. After 14 d of co-culture, the human SGN-like neurons extended their neurites radially toward the explant and made direct contact with the hair cells, as confirmed by the hair cell marker myosin 7a (MYO7A) and human-specific NEFL antibody (Fig. 6**b-d;** Additional file 3**;** Additional file 1**:** Fig. S6c, **d**). In 4 experiments, an average of 8.83% and 3.88% of hair cells were innervated by TUBB3^+^ type I or PRPH^+^ type II human SGN-like neurons, respectively.

While the TUBB3^+^ neurons exclusively innervated a single hair cell, PRPH^+^ neurons formed connections with multiple solute carrier family 26 member 5 (PRESTIN)^+^ mouse outer hair cells (Fig. 6**e;** Additional file 1**:** Fig. S6e, **f**). This observation is consistent with *in vivo* cochlear innervation patterns, where type I SGNs innervate a single inner hair cell while type II SGNs form synapses with several outer hair cells [83]. Similar to the pattern of synaptic connections observed in adult human inner ear tissue (Fig. 6**f**), the co-cultures had (38.8±23.5)% paired pre- and post-synaptic [C-terminal binding protein 2 (CtBP2)^+^ postsynaptic density protein 95 (PSD95)^+^] puncta (Fig. 6**g**, **h**). Due to the culture system (i.e., requiring cell adherence to the dish), the newly formed synapses were not always located at the base of the hair cells, as would be expected *in vivo*, and instead formed on various regions of the cell body. This is consistent with the results of prior studies of cultured inner ear tissue, which have observed the apical repositioning of ribbon synapses [84], [85].

To test if paired CtBP2^+^PSD95^+^ puncta between mouse hair cells and human SGN-like neurons in the co-culture correspond to functional glutamatergic synapses, we performed Ca^2+^ imaging of human SGN-like neurons. This approach leveraged the fact that hair cells from the P5 mouse cochlea spontaneously fire Ca^2+^ spikes, which trigger glutamate release onto SGNs, leading to bursts of APs via synaptic activation [86], [87]. Indeed, on the co-culture of human SGN-like neurons with P5 mouse hair cells, we observed Ca^2+^ transients in the soma of human SGN-like neurons located in the vicinity of hair cells (Fig. 6**i**). To confirm that these Ca^2+^ transients are due to glutamatergic synaptic transmission from hair cells, we applied 6-cyano-7-nitroquinoxaline-2,3-dione (CNQX) and (2 R)-2-Amino-5-phosphonopentanoic acid (AP5), antagonists of AMPA/kainate and NMDA receptors, respectively. The CNQX/AP5 cocktail abolished Ca^2+^ transients (Fig. 6**j-l**), demonstrating that observed Ca^2+^ transients were mediated by glutamate released from spontaneously active hair cells.

Ca²⁺ imaging of human SGN-like neurons cultured alone also showed spontaneous Ca²⁺ transients. However, approximately 70% of these transients were unaffected by glutamatergic receptor blockers (Fig. 6**m-p**). This observation is consistent with intrinsic spontaneous activity seen *in vivo*, known to play an important role in the refinement of auditory circuits before the onset of hearing [88], [89], [90], [91]. Such intrinsic activity may be mediated by hyperpolarization-activated cationic channels or voltage-gated K^+^ channels [92], both of which are expressed in human SGN-like neurons.

To extend these functional assays, we examined c-Fos expression in the co-culture system. Synaptic input triggers Ca²⁺ influx into postsynaptic neurons, activating pathways such as the MAPK/ERK, which drive *c-fos* gene transcription and nuclear c-Fos protein expression [93], [94]. Based on this mechanism, we hypothesized that synaptic activity between hair cells and human SGN-like neurons would induce c-Fos. Indeed, in co-culture, (30.81±13.63)% of human SGN-like neurons expressed c-Fos, whereas this proportion was reduced to (6.60±6.07)% following CNQX/AP5 treatment (Additional file 1**:** Fig. S6g). The human SGN-like neurons cultured alone showed less, but detectable, c-Fos expression (4.92 ± 0.36)%, congruent with less frequent intrinsic spontaneous activity observed in Ca²⁺ imaging.

Together, these findings suggest that human SGN-like neurons are capable of forming functional glutamate-mediated synaptic connections with hair cells in co-culture. This capability is supported by the early expression of AMPA and NMDA receptor subunits (GRIA2–4 and GRIN2B) (Additional file 1**:** Fig. S4e), as well as by previously published *in vivo* functional studies of mouse auditory development [86].

### 3.7 Human SGN-like neurons form functional synapses onto CN neurons

**Fig. 7:**
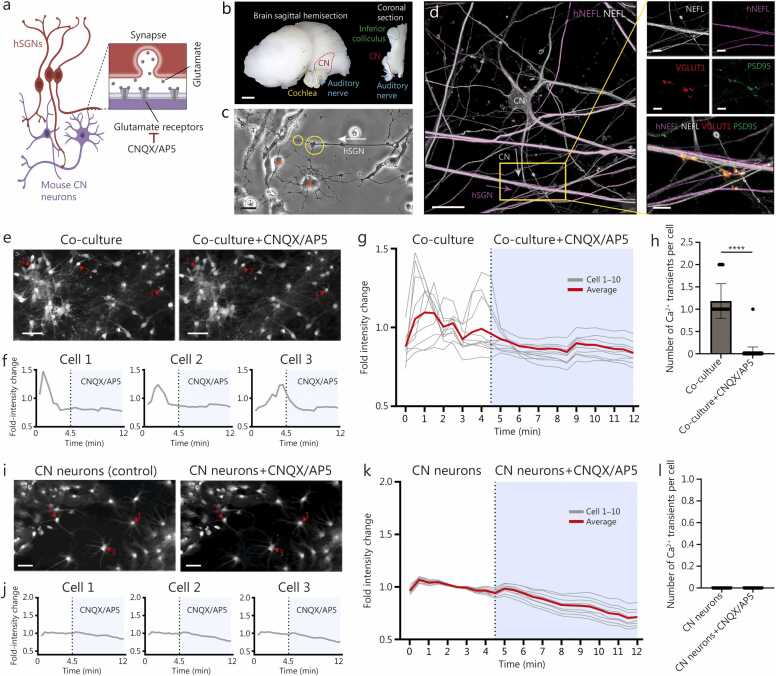
The human SGN-like neurons form functional synaptic connections with CN neurons as demonstrated by Ca^2+^ imaging. **a** A schematic illustrating the co-culture setup of human SGN-like neurons with mouse CN neurons derived from the brainstem. **b** Left: lateral view of a sagittal mouse brain hemi-section (olfactory bulb removed), including the cochlea, auditory nerve, and CN. Right: coronal brain section including the auditory nerve, CN, and inferior colliculus, which is one of several possible auditory pathways located in the midbrain. The inferior colliculus was used as an anatomical landmark for CN location in the coronal plane. Scale bar=1 mm. **c** Bright-field images of P5 mouse CN neurons (red dots) emerging from CN tissue. Yellow circles highlight a connection between multipolar CN neurons and D60 human SGN-like neurons at co-culture D6. The arrow indicates a human SGN-like neuron. Scale bar=20 μm. **d** Co-culture of D78 human SGN-like neurons with P5 mouse CN neurons at D24, immunostained for presynaptic (VGLUT1) and postsynaptic (PSD95) puncta. A human-specific NEFL antibody (hNEFL; purple) labels only human neurons, while a general NEFL antibody (NEFL; gray) labels both mouse and human neurons. Scale bar=20 μm. **e** Ca^2+^ imaging of D108 human SGN-like neurons co-cultured with P5 CN neurons. Left: maximum intensity projection over 4.5 min. Right: maximum intensity projection over 7.5 min after CNQX/AP5 treatment. Scale bar=50 μm. The numerals 1, 2, and 3 denote Cell 1, Cell 2, and Cell 3 used for analysis, respectively. **f** Example Ca^2+^ transients from three CN neurons in (**e**), illustrating Ca^2+^ signal loss after CNQX/AP5. Ca^2+^ signals were normalized to the mean baseline intensity. **g** Representative experiment showing the average relative change in Ca^2+^ signal from 10 CN neurons (P5) co-cultured with D108 human SGN-like neurons, showing loss of signal upon synaptic inhibition. **h** Quantification of the number of Ca^2+^ transients in the co-culture before and after CNQX/AP5 treatment. Data from three different preparations (D61, D63, D108, D129, D131, and D213) for a total of 55 CN neurons. **i-l** As in (**e-h**), but for CN neurons-only culture (P5, control). The numerals 1, 2, and 3 in panel i denote Cell 1, Cell 2, and Cell 3 used for analysis, respectively (**i**). Scale bar=100 μm. No discernible Ca^2+^ transients were observed under these conditions. *n*=20 CN neurons (P5), with or without CNQX/AP5 treatment. ^⁎⁎⁎⁎^*P*<0.0001 (the Mann-Whitney test). D. Day; Ca^2+^. Calcium ion; NEFL. Neurofilament light chain; CN. Cochlear nucleus; P. Postnatal day; SGN. Spiral ganglion neuron; CNQX. 6-cyano-7-nitroquinoxaline-2;3-dione; AP5. D(-)-2-amino-5-phosphonopentanoic acid; VGLUT1. Solute carrier family 17 member 7; PSD95. Postsynaptic density protein 95.

In mammals, SGNs directly project to the CN of the brainstem, the first brain structure of the central auditory pathway [95]. We tested whether the human SGN-like neurons could project to and/or attract neurons from dissected mouse CN (Fig. 7**a**). As early as D6 in co-culture, neurons were observed to emerge from the CN explant to form connections with the human SGN-like neurons (Fig. 7**b, c;** Additional file 1**:** Fig. S7a). The neuron types can be distinguished by their unique morphology, as CN neurons are multipolar while the human SGN-like neurons are bipolar, and by staining with human-specific and human- and mouse-reactive NEFL antibodies. The CN neurons exhibited several different shapes, including bushy and stellate cell-like cells, likely corresponding to anteroventral and posteroventral CN, and fusiform cell-like cells likely corresponding to the dorsal CN [96] (Additional file 1**:** Fig. S7b). In this system, we observed presynaptic markers [synaptophysin (SY)^+^ and VGLUT1^+^] associated with human SGN-like neurites and a post-synaptic marker (PSD95^+^) associated with CN neurites in close proximity to each other, suggesting that human SGN-like neurons form synapses with CN neurons (Fig. 7**d;** Additional file 1**:** Fig. S7c).

As with mouse hair cell to human SGN-like neuron co-culture, we performed Ca^2+^ imaging to determine if human SGN-like neurons make functional synapses onto CN neurons. Prior to co-culturing, human SGN-like neurons were labeled with Dil to distinguish them from CN neurons. In the co-culture, Ca^2+^ transients observed in CN neurons were abolished in the presence of CNQX and AP5 (Fig. 7**e-**h), suggesting their synaptic origin. In contrast to co-culture, Ca²⁺ imaging of CN neurons cultured alone revealed no Ca²⁺ transients (Fig. 7**i-l**), suggesting that glutamatergic contacts between CN neurons, if they exist, do not result in significant depolarization to trigger Ca^2+^ transients. Consistent with these findings, (10.78±5.94)% of CN neurons in co-culture expressed c-Fos, whereas expression was reduced to (1.91±0.47)% following the CNQX/AP5 treatment (Additional file 1**:** Fig. S7d). CN neurons cultured alone (control) exhibited almost no detectable c-Fos expression (0.96±0.70)%.

Together, these findings indicate that the CNQX/AP5-sensitive Ca²⁺ activity in mouse CN neurons when co-cultured with human SGN-like neurons is likely due to glutamatergic input from human SGN-like neurons into CN neurons.

## 4 Discussion

Although hearing loss disables over 5% of the world’s population [97], there are currently no effective pharmaceutical or cellular therapies approved for sensorineural hearing loss. Discovery and testing of such therapies can be propelled via reliable and physiologically relevant *in vitro* human inner ear models. Here, we present a robust differentiation protocol for generating human SGN-like neurons from hiPSCs, designed to mimic the signaling cues from surrounding tissue present during human auditory neuron development *in vivo*. We carefully interrogated the lineage, molecular signatures, gene and protein expression, and functional properties of the resulting cells to increase confidence that they possess SGN characteristics. This approach enabled a level of validation and in-depth analysis of human SGN-like neurons previously possible only in rodent models [69], [71].

Our differentiation protocol resulted in neurons exhibiting electrophysiological properties previously reported in *ex vivo* SGNs [71], [72], and evidenced by the presence of Na^+^- and K^+^-mediated currents, hyperpolarized resting potentials, overshooting APs, and firing patterns consistent with mature SGN subtypes (MA and UA). These changes are consistent with the increase in expression of mRNA encoding for various Na^+^ and K^+^ channels. Moreover, the passive and active properties of human SGN-like neurons described here parallel neuronal developmental mechanisms previously described for central neurons in various brain regions [98], [99]. Notably, the human SGN-like neurons generated in this study elicit overshooting APs and firing patterns that closely resemble *ex vivo* recordings of mature SGNs [71], [72].

In addition to maturing intrinsic excitability properties, human SGN-like neurons have the necessary molecular machinery to form functional synapses. The expression of mRNA encoding various AMPA and NMDA receptor subunits supports their capacity to establish glutamatergic synapses with both hair cells and CN neurons. These synapses contain functional CNQX- and AP5-sensitive AMPA and NMDA receptors, similar to what is shown in *ex vivo* work in animal models [86], [100], [101]. This demonstrated ability to functionally integrate with both pre- or post-synaptic partners represents a critical milestone toward establishing physiologically accurate models of human hearing.

Our methodology represents a fundamental improvement over prior protocols for the generation of human SGN-like neurons from hiPSCs, particularly because of the high purity of inner ear-related cells achieved, the in-depth description of cell type identities to inform future studies, and simplification of the culture system to maximize its utility (Additional file 4**: Comparison of methodological approach**). Our protocol allowed the differentiation of hiPSCs into early inner ear lineage (i.e., otic vesicle- and CNC-related) cells at >99% efficiency on D25, and into human SGN-like neurons and surrounding (i.e., glia and mesenchymal) cells at >98% efficiency on D60. Although additional studies are needed, our findings suggest that these human SGN-like neurons are developing along the trajectory that enables their relaying of tonotopic information. This is supported by two observations. First, we identified at least two subpopulations of type II SGN-like neurons (TH^+^PRPH^+^ and TH^-^PRPH^+^). *In vivo*, TH^+^ type II SGNs are known to be distributed along a tonotopic gradient, with a higher proportion at the cochlear apex compared to the base [102]. Second, human SGN-like neurons formed synaptic connections with multiple types of CN neurons, including bushy, stellate, and fusiform cells, which likely originate from anteroventral, posteroventral, and dorsal regions of CN, respectively. These findings suggest that human SGN-like neurons may have a capacity to innervate CN in a manner consistent with tonotopic organization. The unprecedented maturity of the human SGN-like cells, roughly equivalent to SGNs at the second trimester of gestation, is likely due to their co-generation with Schwann cells, satellite glia, and periotic mesenchymal cells whose roles may have been underestimated in prior protocols. *In vivo*, these cell types reciprocally interact with SGNs during inner ear development and are essential for their maturation [11], [103], [104], [105]. Thus, the strategy of co-generating human SGN-like neurons with “neighboring” cells likely enabled this protocol’s high efficiency and reproducibility, and the unprecedented maturity of target human SGN-like neurons.

This system could offer powerful cellular models to decipher the precise mechanisms underlying hearing disorders and enable future targeted therapies. For example, our high-efficiency, monolayer differentiation protocol can be a reliable source of human SGN-like neurons suitable for drug screening. Although 3D organoids have been spotlighted for this purpose due to their multicellular structure, they are less compatible with high-throughput screening. In contrast, our two-dimensional (2D) culture system provides benefits for easier scaling, real-time monitoring, functional tests, and quality control in a realistic way, as has already been demonstrated using dissociated mouse SGNs [106]. These features are particularly valuable during drug screening when testing thousands of compounds in parallel.

Given the repeated failure of clinical trials for hearing loss therapies that initially demonstrated promising results in animal models, a major strength of our platform is its use of human-derived cells, thereby improving the potential for clinical translation. A particularly compelling near-term application is the concept of an engineered biopsy of the inner ear using a patient’s own hiPSCs. This strategy could be especially valuable considering the large number of genes associated with hearing loss, the current inability to access living inner ear tissue for routine biopsy, and the fact that the histopathology of the human cochlea has been described for only about one-tenth of known deafness-causing mutations [107]. In addition, our human SGN-like neurons may serve as a foundation for future cell-based therapies. Although further studies will be required to determine whether these neurons can integrate into existing neural circuits and restore function, the present work demonstrates that they can form functional synaptic connections with both peripheral and central targets, which would be an essential prerequisite for the restoration of hearing following neuronal damage. Clinical translation of such an approach would require the development of minimally invasive round window injection techniques when aimed at reestablishing biological hearing. Importantly, therapeutic applications may be realized sooner in the context of cochlear implants. As the hearing ability and speech recognition in cochlear implant users are thought to correlate with the number of surviving SGNs [108], [109], patient-specific SGNs might be used co-therapeutically with cochlear implants to achieve better auditory outcomes. The current protocol provides an ideal platform for testing this translational application in the future, as well as addressing other important questions in otologic research.

Although this study establishes a robust protocol for generating SGN-like neurons from hiPSCs, arguably the closest to their *in vivo* counterpart to date, several limitations remain before the platform can be advanced toward translational relevance. First, a more thorough exploration of communication between human SGN-like neurons and their partner cells is necessary. In this study, we assessed cell-cell communication using synaptic marker staining and Ca^2+^ imaging in co-culture systems. However, the newly formed synapses were not always positioned at the base of the hair cells, as is observed *in vivo*, and long-term synaptic activity remains to be demonstrated. In addition, because Ca^2+^ imaging relied primarily on spontaneous activity, only a limited proportion of cells were activated. Future approaches, such as picospritzer, multi-chamber microfluidic system (to stimulate distinct populations with KCl), or optogenetic tools (e.g., channel rhodopsin expressing cells), may enable more controlled stimulation. Importantly, systematic testing of bidirectional signaling in co-cultures involving mouse hair cells, human SGN-like neurons, and mouse CN neurons will be critical before moving to *in vivo* studies. Second, functional characterization of human SGN and glia interactions is needed. While our immunostaining demonstrated satellite glia-like cells surrounding human SGN-like neurons and Schwann cell-like cells wrapping axons, functional assays were not performed. Perturbation or co-culture assays could elucidate the roles of glia in synapse formation, axon guidance, and neuronal maintenance. For example, assessing SGN neurite growth toward mouse hair cell targets while selectively ablating Schwann cells (e.g., with fluoroacetate) could provide valuable insights, with neurite length as a primary readout. Third, while our current 2D culture system offers a practical platform, particularly for drug screening, transitioning to 3D organoids or scaffold-based systems will be important to more closely recapitulate the native inner ear environment. Such approaches could better mimic the mechanical, biochemical, and structural cues that shape auditory neuron development and function *in vivo*. Finally, *in vivo* survival and transplantation studies will be required to determine the clinical potential of hiPSC-derived SGN-like neurons. Initial experiments in healthy animal models should assess long-term survival, integration into cochlear circuits, and synapse formation with both hair cells and CN neurons. Subsequent transplantation into auditory neuron injury models, followed by functional assessments such as auditory brainstem responses and distortion product otoacoustic emissions, will provide essential evidence of therapeutic potential. Together, these directions highlight both the promise of this platform and the critical next steps needed for its translation into future therapies.

## 5 Conclusions

This study presents a reliable and scalable 2D differentiation protocol for generating human SGN-like cells from hiPSCs, recapitulating key developmental stages of the human inner ear. The resulting neurons exhibit structural, molecular, and functional characteristics similar to primary SGNs found *in vivo*, including the ability to form functional glutamatergic synapses with both peripheral and central auditory targets. Importantly, this platform offers several advantages for translational applications: it enables access to otherwise unattainable human auditory neurons without compromising hearing, supports functional analysis of patient-specific genetic mutations, and is compatible with high-throughput drug testing. Given that hearing loss is a major cause of disability among both civilian and military populations, our system provides a practical foundation for accelerating therapeutic discovery and advancing personalized regenerative strategies.

## Abbreviations

APsAction potentials
BDNFBrain-derived neurotrophic factor
Ca^2+^Calcium ion
CDMChemically defined media
CmMembrane capacitance
CNCochlear nucleus
CNCCranial neural crest
DDay
EMTEpithelial-mesenchymal transition
HBSSHank’s Balanced Salt Solution
hiPSCsHuman induced pluripotent stem cells
IGF-1Insulin-like growth factor-1
ISIInter-spike interval
MAMulti-spike accommodating neurons
MPZMyelin protein zero
MYO7AMyosin 7a
NEFLNeurofilament
NEUROD1Neurogenic differentiation 1
NGFRNerve growth factor receptor
NT3Neurotrophin-3
ONPOtic neurosensory progenitor
PPostnatal day
PAX2Paired box 2
PBSPhosphate-buffered saline
PRPHPeripheral neuronal marker peripherin
scRNA-seqSingle-cell RNA sequencing
SDStandard deviation
SHHSonic hedgehog
SGNsSpiral ganglion neurons
SOX2SRY-box transcription factor 2
UMAPUniform manifold approximation and projection
UAUnitary-spike accommodating neurons
TUBB3Tubulin beta 3 class III
VGLUT1Vesicular glutamate transporter 1
WWeeks of gestation

## Ethics approval and consent to participate

All procedures involving human subjects were conducted in accordance with the Declaration of Helsinki. Research involving human induced pluripotent stem cells was reviewed by the Human Studies Committee of the Massachusetts General Brigham Institutional Review Board and granted approval on 24 May 2018 (14-148H) and 2 November 2018 (16-066H). Research involving human vestibular tissue obtained from patients was reviewed by the Massachusetts General Brigham Institutional Review Board and determined to be exempt from approval (2020P003329) on 2 December 2020. All procedures involving animals were approved by the Institutional Animal Care and Use Committees of Massachusetts Eye and Ear Infirmary in Boston, MA, and of Stanford University in Stanford, CA (approved protocol ID: 33998). All procedures with animals were conducted under the National Academies of Sciences Guide for the Care and Use of Laboratory Animals (8th edition, NAS Press, USA).

## Funding

This work was supported by the National Institutes of Health (NIH) grants (U24 DC020857 to KMS, U01 DC019370 to RH, and R01 DC018353 to AET), the NIH-NIDCD Division of Intramural Research fund (DC000094-01 to RH), the Hearing Health Foundation (to RH), the Pew Latin American Fellows Program in the Biomedical Sciences (to LGV), the Remondi Foundation (to KMS), the Bertarelli Foundation Endowed Professorship (to KMS), and Jennifer and Louis Hernandez (to KMS).

This research was supported in part by the Intramural Research Program of the National Institutes of Health (NIH). The contributions of the NIH author(s) are considered Works of the United States Government. The findings and conclusions presented in this paper are those of the author(s) and do not necessarily reflect the views of the NIH or the U.S. Department of Health and Human Services.

## Competing interests

The authors declare that they have no competing interests.

## Data Availability

All datasets supporting the conclusions of this article are included within the article and its additional files. Additional data are available from the corresponding author upon reasonable request. The scRNA-seq dataset supporting the conclusions of this article is available in the Gene Expression Omnibus data repository under accession code: GSE252741. It is also accessible via the Gene Expression Analysis Resource (gEAR) [110] portal at: https://umgear.org/p?l=sk8aNeuron.

## Appendix A Supplementary material

Supplementary material Additional file 1. Methods. Fig. S1 Higher seeding density on D11 promotes the formation of ONP-like cells and subsequent neuronal development. Fig. S2 Maturation and myelination of human SGN-like neurons. Fig. S3 Quality control and scRNA-seq data. Fig. S4 Differential expression of genes related to neurotransmission. Fig. S5 Firing properties, AP parameters at rheobase, and voltage responses to hyperpolarizing steps in type I human SGN-like neurons. Fig. S6 Co-culture of hiPSC-derived human SGN-like neurons and mouse denervated hair cells. Fig. S7 Co-culture of hiPSC-derived human SGN-like neurons and mouse CN neurons

Supplementary material Additional file 2. Medium composition

Supplementary material Additional file 3. Antibody list

Supplementary material Additional file 4. Protocol novelty

Supplementary material Additional file 5. Gene expression on D25

Supplementary materialAdditional file 6. Gene expression on D60

Supplementary materialAdditional file 7. Electrophysiological characterization
