# Supplementary material for: Engineering of functional auditory neurons from human induced pluripotent stem cells
Source: Mil Med Res. 2026 Apr 15;13(1):100008. doi: 10.1016/j.mmr.2026.100008 (PMC13127150; doi:10.1016/j.mmr.2026.100008)
Supplement: Supplementary file 1 — Supplementary material Additional file 1. Methods. Fig. S1 Higher seeding density on D11 promotes the formation of ONP-like cells and subsequent neuronal development. Fig. S2 Maturation and myelination of human SGN-like neurons. Fig. S3 Quality control and scRNA-seq data. Fig. S4 Differential expression of genes related to neurotransmission. Fig. S5 Firing properties, AP parameters at rheobase, and voltage responses to hyperpolarizing steps in type I human SGN-like neurons. Fig. S6 Co-culture of hiPSC-derived human SGN-like neurons and mouse denervated hair cells. Fig. S7 Co-culture of hiPSC-derived human SGN-like neurons and mouse CN neurons [file mmc1.pdf]

Methods

Human induced pluripotent stem cell (hiPSC) culture

hiPSC lines SK8-A, generated in our laboratory [1], and UCSD112i-2-11 (UCSD), purchased from WiCell (Madison, WI, USA), were used before passage number 50. Cells were maintained on Matrigel human embryonic stem cell (hESC) qualified matrix (Cat# 354277, Corning, USA) in mTeSR1 (Cat# 85850, StemCell Technologies, Canada) or mTeSR plus medium (Cat# 100-0276, StemCell Technologies, Canada) supplemented with 1× penicillin-streptomycin (Cat# 15140122, Gibco, USA). Cells were passaged at around 80% confluency, and hiPSC colonies were treated with ReLeSR (Cat# 100-0484, StemCell Technologies, Canada) for detaching by tapping the side of the plates. Detached cell clumps were plated on new Matrigel-coated 6-well plates. The medium was replenished every day (mTeSR1) or every other day (mTeSR plus). Detailed information about the origin and characterization of cell line UCSD is available at: https://www.wicell.org/home/stem-cells/catalog-of-stem-cell-lines/ucsd112i-2-11.cmsx?closable=true.

The culture method described in this study was replicated by two independent investigators in separate locations [M.J. (Massachusetts and California) and Petra Stojkovic (Massachusetts)] using the cell lines SK8-A and UCSD. Thus, we confirmed that the success of this protocol is not limited by a person, cell lines, or place.

Human vestibular tissue

To examine the morphology and key protein expression of mature human SGNs, fresh inner ear vestibular tissue was collected during surgical labyrinthectomies and translabyrinthine resections of vestibular schwannomas (*n*=2) occurring as part of routine clinical management. The notation regarding this tissue, as reported by the surgeon at the time of excision, was “vestibular end organs” which together include

saccular and utricular maculae and cristae of the semicircular canals; further details as to the specific organ were not available.

Dissociation of human SGN-like cells for scRNA-seq

To dissociate the samples generated from hiPSCs SK8-A and UCSD into single cells, randomly selected wells containing the cells on differentiated D25 (*n*=3 for both lines), D60 (*n*=6), D90 (*n*=6), and D120 (*n*=6) were pooled. In brief, collected cells were incubated with TrypLE Select for about 5 min in a 37 °C incubator until detachment of the edge of cells was observed. After removing TrypLE Select from the plate, 10% fetal bovine serum in the Neurobasal medium was added and gentle pipetting using p1000 tips until no visible cell aggregation. The suspension was filtered through a 100 μm cell strainer to eliminate any chunks of cell aggregates. Additionally, Debris Removal Solution (Cat# 130-109-398, Miltenyi Biotec, Germany), a density gradient reagent, was used to remove debris from viable cells. Then, cells were resuspended in 1× Dulbecco's PBS (Cat# 14190144, Gibco, USA) containing 0.04% bovine serum albumin (Cat# A8412, Sigma, USA). Cell viability and live-cell counting were determined by manual counting using a hemocytometer and cell BioRad TC20. The final cell concentration was 1000 cells/μl with cell viability above 80%.

scRNA-seq cDNA library preparation and sequencing

Single-cell 3' RNA-seq experiments were conducted using the Chromium single-cell system (10× Genomics, USA) and the NexSeq 2000 (Illumina, USA). The dissociated cells were added to a single-cell master mix (targeting 10,000 cells), following the Chromium Single Cell G000183 Chromium Single Cell3' version 3 user guide, revision C (10× Genomics, USA). Along with the single-cell gel beads and oil partitioned in separate wells of a Single Cell B Chip, the single-cell reaction mixture was loaded into the Chromium Controller for Gel Bead-in-Emulsion generation and barcoding, followed by cDNA synthesis and library preparation. At each step, the quality of the cDNA and library was examined by

Tapestation 4200 (Agilent Technologies, USA). The resulting library was sequenced in a custom program for 28-bp plus 91-bp paired-end sequencing on an Illumina NextSeq 2000 to a reading depth of more than 30,000 reads per cell.

scRNA-seq data analysis

The 10× Genomics Cell Ranger 2.1.0 pipeline (http://support.10xgenomics.com/) was used to process raw sequence data. In brief, Cell Ranger with bcl2fastq (https://support.illumina.com/) was used to demultiplex raw base sequence calls generated from the sequencer into sample-specific FASTQ files. The FASTQ files were then aligned to the reference genome with RNA-seq aligner Spliced Transcripts Alignment to a Reference (STAR) software. The aligned reads were traced back to the individual cells, and the gene expression level of individual genes is quantified based on the number of unique molecular indices detected in each cell. Filtered gene-cell barcode matrices were generated by Cell Ranger for further analysis. Cells with fewer than 200 unique genes identified and cells with more than 6000–8000 total RNA molecules detected were all removed from the analysis. In addition, cells with higher than 10%–20% mitochondrial reads were filtered and removed from the analysis. To detect doublets, DoubletFinder [2] with standard parameters was used, and the doublet rate was predicted based on the number of cells captured (3.2%–7.2%).

After removing low-quality cells, the gene expression levels for each cell were normalized by the total number of unique molecular indices in the cell and multiplied by a scaling factor of 10,000. After log-transformation, we used Seurat v.4.0.4-5.0.0 for cell clustering using principal component analysis on highly variable genes. Cell cluster visualization was done in uniform manifold approximation and projection (UMAP) space, which offers preservation of the data's global structure. The gene markers for each cluster were identified through differential expression analysis by comparing cells in the cluster to all other cells. Cell cluster identities were manually defined with the cluster-specific marker genes. To analyze the subgroups of clusters, we applied functions "subset" from Seurat. The expression of a range

of pan-neuronal markers, such as tubulin beta 3 class III (*TUBB3*) and sodium/potassium-transporting ATPase subunit beta-1 (*ATP1B1*), was used to subset neuron clusters marked as red dot circles. The R package ggplot2 was used to plot the average gene expression. Violin plots and feature plots were used to visualize specific gene expressions across clusters and different sample conditions. Integration via the harmony algorithm [3], which has been shown to adequately remove batch effects while conserving biological variation, was used to compare D25 and D60 SGN-like cells to embryonic day (E) 13.5 [4] and E14–postnatal day (P) 1 [5] mouse datasets, respectively.

Immunocytochemistry and imaging

Cells grown on coverslips were fixed with 4% paraformaldehyde (Cat# AAJ19943K2, ThermoFisher Scientific, USA) for 10 min at room temperature. For permeabilization, cells were washed 3 times with PBS (Cat# 14080-055, Gibco, USA) and incubated in PBST, which is 0.1% Triton X-100 (Cat# T8787, Sigma, USA) in 1× PBS solution, for 10 min at room temperature. Unspecific binding was blocked with 5% normal horse serum (Cat# ab7484, Abcam, USA) or 5% goat serum (Cat# PCN5000, Gibco, USA) in PBST for 1 h. Samples were then incubated overnight at 4 °C with specific primary antibodies diluted in 1% bovine serum albumin (Cat# A9647, Sigma, USA) in PBST, washed 3 times with PBS, and incubated with secondary antibodies in PBST (**Additional file 3**). Vectashield (Cat# H1000, Vector Laboratories, USA) with DAPI (Cat# 4083, Cell Signaling, USA) was used to mount the samples and visualize cellular nuclei. Negative control experiments without the primary antibodies were processed in parallel. Microscopy was performed using a Leica SP8 confocal microscope (Leica Microsystems, Germany) or ZEISS LSM 880 (Carl Zeiss AG, Germany).

For co-culture staining, a similar protocol was used. Briefly, coverslips on hiPSC-derived SGNs and mouse explants were rinsed in PBS, fixed with 4% paraformaldehyde in PBS for 20 min, washed with PBS, and blocked in a blocking buffer consisting of 5% normal horse serum with 0.5% Triton X-100 at room temperature on a horizontal shaker for 30 min. Samples were then incubated overnight at 4 °C with

specific primary antibodies diluted in 1% normal horse serum in 0.5% Triton X-100, washed 3 times with PBS, and incubated with secondary antibodies in PBST for 90 min (**Additional file 3**). Specimens were imaged with a Leica SP8 confocal microscope (Leica Microsystems, Germany). After taking an overview of the specimen at 20× magnification, images were reconstituted in a 3D mode to count hair cells and find connections between mouse hair cells and SGN neurites derived from hiPSCs. Then, the area of synaptic connection was visualized by 63× with 2.4× digital zoom at a Z-step-size of 0.3 μm. To avoid imaging with overlapping or close wavelengths simultaneously, samples were sequentially scanned.

Electrophysiological recordings

Whole-cell electrophysiological recordings were made from human SGN-like neurons generated from two different hiPSCs. Recorded neurons were maintained for D88–95 or D120–234 *in vitro*. During recordings, neurons were continuously superfused in artificial cerebrospinal fluid containing (in mmol/L): 125 NaCl, 2.5 KCl, 1.25 NaH_2PO_4, 25 $NaHCO_3$, 25 D(+)-glucose, 1 $MgCl_2$, and 2 $CaCl_2$, buffered with 5% carbogen. Neurons were visualized under infrared differential interference contrast (IR-DIC) with a microscope (Scientifica, UK; Olympus Americas, USA) using an Orca Flash 4.0 CMOS digital camera C13440 (Hamamatsu Photonics, Japan). Patch pipettes with resistances of 3–4 MOhm were pulled using a Flaming/Brown Micropipette Puller P-97 (Sutter Instruments, USA) and filled with intracellular solution containing (in mmol/L): 115 K-gluconate, 7 KCl, 10 N-(2-hydroxyethyl)piperazine-N'-(2-ethanesulfonic acid) (HEPES), 0.05 ethyleneglycol- bis(β-aminoethyl)-N,N,N',N'-tetraacetic acid (EGTA), 2 Na_2-ATP, 2 Mg-ATP, and 0.5 Na_2-GTP (pH adjusted to 7.3 with KOH; osmolarity 285 mOsm). For post-hoc identification, 0.1%–0.3% biocytin was loaded into cells via patch-pipettes. Biocytin was visualized after conjugation with streptavidin-Alexa Fluor 488.

Recordings were made at room temperature (22 ˚C) and liquid junction potentials were not corrected. Series resistance (Rs), membrane resistance, capacitance, and time constant were calculated from a 10 mV hyperpolarizing step with a duration of 100 ms applied in voltage-clamp configuration. Whole-cell

recordings were obtained with compensated pipette capacitance and Rs (60%) during the experiment. Analyzed data only included recordings with Rs <30 MOhm. Data were acquired and low-pass filtered at 3 kHz with a Multiclamp 700B amplifier (Molecular Devices, USA), in combination with custom software written in Matlab (Mathworks, USA) based on scripts developed in the Sabatini (https://github.com/bernardosabatinilab) and Sanes laboratories. A USB NI-6343 digital-to-analog converter (National Instruments, USA) was employed to digitize data at 10 kHz. The analysis was performed offline with custom-written Matlab routines.

The intrinsic neuronal properties and action potential (AP) features were determined by applying a series of hyperpolarizing and depolarizing steps while in current-clamp configuration. For voltage-clamp recordings, neurons were kept at –70 mV, and voltage stepped from –100 mV to 40 mV in 10 mV increments. Putative potassium currents were calculated as the difference in current amplitude between the last 10 ms of the stimulation protocol and the baseline amplitude after leak current was subtracted. Putative sodium currents were calculated as the difference in current amplitude between the inflection point after the first capacitive transient and the negative current. For current-clamp recordings, current injections from –80 pA to 400 pA in steps of 20 pA were applied. Rheobase was defined as the minimal depolarizing current to elicit an AP. If the rheobase was not reached before 400 pA, current injections from –80 pA to 800 pA were applied. AP properties were analyzed both at rheobase and at the maximum current step to which steady APs were elicited. Neurons were classified as multi-spike accommodating neurons (MA) when 3 or more APs were evoked, and as unitary-spike accommodating neurons (UA) when fewer than 3 APs in a given current injection step were observed across the whole stimulation protocol. AP threshold for the first AP within a step was defined as the membrane potential value at the point before the peak of each AP in which dV/dt was 7.5% of the maximum dV/dt. The latency to the first AP during a current injection step was calculated as the time between the start of the stimulus and the time of the AP threshold, which was considered the onset of the AP. The averaged inter-spike interval (ISI) was measured

as the mean difference in onset times between APs within a step, in MA neurons only. For each current injection step, the average half-width was determined as the mean of the width of all APs in response to that current step at 50% of the membrane potential value between the threshold and the peak of each AP. Average AP height was determined as the mean of the difference between the membrane potential value at the peak of each AP and the value at the subsequent trough.

Co-culture with mouse hair cells

Cochlear explants were obtained from P3–6 CBA/CaJ or NOD/SCID wild-type mice (Jackson Laboratory; n=30). At this neonatal stage, sex cannot be reliably determined; therefore, both sexes were included. Dissection of cochlear explants was performed largely as previously described [6]. Briefly, pups were decapitated, and the external auditory canals were incised with a scalpel blade. The skin was folded anteriorly to expose the cranium. The cranium was opened along the sagittal suture using a #15 scalpel blade. A vertical cut was made posterior to the orbits to remove and discard the snout. The forebrain, cerebellum, and brainstem were removed through blunt dissection using #4 forceps. The cranium was placed into a plastic Petri dish filled with cold HBSS, and the cochlea was completely exposed, located adjacent to the stapedial artery (a tortuous artery within the temporal bone). The cochlea was then bluntly separated from the temporal bone using #4 forceps. The cochlea was carefully dissected from the vestibular system, and the cochlear otic capsule (typically cartilaginous at this age) was dissected with #4 forceps.

The spiral ligament, adherent to the organ of Corti, was separated from the rest of the cochlea and the modiolus, using a micro knife or two forceps, starting from the base and moving toward the apex. The organ of Corti was carefully dissected into a more apical and a more basal part, containing sensory hair cells and dendrites of SGNs. The tectorial and Reissner's membranes and cell bodies of SGNs were removed. In an additional set of experiments, cochlear explants from P4 CBA/CaJ and Thy1-GFP BL6 (Jackson Laboratory) were denervated to dissect the sensory epithelium (organ of Corti, containing hair

cells) from the spiral limbus (containing neurites of SGN), using microdissection techniques previously described [7].

After isolating the cochlear explants or denervated hair cells, they were transferred onto a 12 mm diameter coverslip (Cat# GG12-1.5-oz, Neuvitro, USA) on which hiPSC-derived cells were cultured. The explants were placed approximately 500 μm away from a cluster of human SGN-like cell somas, allowing space for the neurites of human SGN-like neurons to extend toward the mouse hair cells. To prevent the explants from detaching, only a minimal amount (200–250 μl) of SGN differentiation medium (D25) was added, just enough to cover the explants. The medium was replenished approximately 24 h later, after the explants had attached to the coverslip. The medium was changed every 2–3 d based on its color (indicating pH changes) and evaporation rate. The cultures were monitored daily under a bright-field inverted microscope to assess the cells' condition and the potential connection between mouse hair cells and human SGN-like neurons. The co-cultures were maintained in a 37 °C, 5% CO_2 incubator for up to 14 d and were fixed with 4% paraformaldehyde for a downstream immunostaining analysis.

To quantify synaptic puncta, we counted all C-terminal binding protein 2 $(CtBP2)^+$/postsynaptic density protein 95 $(PSD95)^-$, $CtBP2^-/PSD95^+$, and $CtBP2^+/PSD95^+$. All these combined numbers are denominators for calculating the percentage of paired synaptic puncta. Co-cultures were maintained for up to 14 d. As a control, cochlea explants or denervated hair cells were cultured in isolation (n=6, 3 mice for each) in the same way and duration.

Co-culture with the mouse cochlear nucleus (CN)

CN tissue was collected from P3–6 pups (n=17) and 8-week-old CBA/CaJ mice (n=1) of either sex. Pups were anesthetized by inducing hypothermia and promptly decapitated when unconscious. The head was placed in a 60 mm Petri dish containing 70% ethanol, and the skull was exposed with a medial-sagittal incision on the scalp with a #15 blade. After cutting the external auditory canals bilaterally, the cranium

was opened along the sagittal suture from anterior to posterior. The cranial incision was then deepened ventrally toward the base of the skull. The snout was removed with a coronal cut posterior to the orbits, and two halves of the cranium were placed in a 60 mm Petri dish containing ice-cold HBSS (Cat# 14025092, Gibco, USA). The forebrain, cerebellum, and brainstem were gently removed from the skull halves by blunt dissection with #5 forceps and transferred to a dish containing fresh ice-cold HBSS. The brainstem was then separated from the cerebral and cerebellar hemispheres. The cochlear nuclei are identified by their relation to several anatomical landmarks on the lateral portion of the brainstem: a) the auditory nerve stump is ventral, b) the inferior cerebellar peduncle is dorsal, and c) the sulcus of the anterior-inferior cerebellar artery is posterior to the structure of interest. A 150–250 μm thick parasagittal slice of the cochlear nuclei is made with a 45° stab microsurgical knife (PE3045, Oasis, USA) following the structure of the nuclei. The sample was subsequently cut into smaller 150 μm×150 μm×150 μm fragments. Some of the samples were pipetted using 200 μl tips for further dissociation.

Cochlear nuclei explant pieces were placed onto 12 mm diameter coverslips containing human SGN-like cells. Typically, each CN explant was divided into three portions and placed on three separate coverslips containing human SGN-like cells. The co-cultured human SGN-like cells and CN were kept in SGN differentiation medium (D25) in an incubator at 37 °C and 5% CO_2. Two days after co-culture, unattached cells or debris from the CN explants were carefully removed, and the culture medium was replenished. The cultures were monitored daily under a bright-field inverted microscope to assess the cells' condition and the potential connection between mouse CN neurons and human SGN-like neurons. Half of the medium volume (approximately 250 μl) was replaced every three days, and the co-cultures were maintained for up to 28 d before being processed for immunofluorescence imaging.

Calcium ion (Ca^{2+}) imaging

Ca^{2+} imaging with fluorescent Ca^{2+} dyes was performed to evaluate the synaptic dependence of spiking activity of human SGN-like neurons or CBA/CaJ mouse CN neurons (P5 or P6) co-cultured with CBA/CaJ

mouse hair cells (P5). Cells were labeled with 10 μmol/L Cal-520 AM (Cat# ab171868, Abcam, USA) in artificial perilymph supplemented with 25 μmol/L Sulfinpyrazone (Cat# S9509, Sigma, USA) and 0.1% Pluronic F-127 (Cat# P6866, Thermo Scientific, USA). The labeling was performed at 37 °C for 90 min. The artificial perilymph solution was freshly prepared, containing (in mmol/L): 144 NaCl (Cat# S7653, Sigma, USA), 5.8 KCl (Cat# P3911, Sigma, USA), 0.7 NaH_2PO_4 (Cat# S8282, Sigma, USA), 10 HEPES (Cat# H3375, Sigma, USA), 0.9 $MgCl_2·6H_2O$ (Cat# M0250, Sigma, USA), 1.3 $CaCl_2·2H_2O$ (Cat# C5080, Sigma, USA), and 5.6 D-glucose (Cat# G7021, Sigma, USA). The pH of the solution was adjusted to 7.4.

Images of fluorescent neurons were captured every 30 s on a Zeiss Axiovert A1 inverted fluorescence microscope. This temporal resolution was sufficient to discern spiking-dependent Ca^{2+} transients and whether they were downstream of synaptic transmission. Baseline Ca^{2+} fluorescence was recorded for 4.5 min before the addition of 10 μmol/L 6-cyano-7-nitroquinoxaline-2,3-dione (CNQX) (Cat# 1045, Tocris, UK) and 50 μmol/L D-(-)-2-amino-5-phosphonopentanoic acid (D-AP5; Cat# 0106, Tocris, UK); imaging in CNQX/D-AP5 was for 7.5 min. Ca^{2+} fluorescence intensity of each human SGN-like neuron or mouse CN neuron was measured with ImageJ software as the average pixel brightness over the soma of each neuron. The fold change, presented on the y-axis of the graphs, was calculated as the ratio of Ca^{2+} fluorescence intensity of each cell to the average baseline intensity.

Statistical analysis

Unless the purpose was solely for structural observation, immunostaining was quantified using ImageJ and reported as the proportion of cells positive for the stain. All immunostaining images are representative of at least 3 replicates, except for those involving human inner ear tissue, which used 2 technical replicates due to scarcity of the tissue.

Electrophysiology recordings were obtained by combining data of 4 and 3 independent experiments with cell lines D88–95 and >D120 SK8-A, respectively, and 1 and 4 independent experiments with cell

lines D88–95 and >D120 UCSD, respectively. Statistical analysis of electrophysiological parameters, including membrane time constant, rheobase, latency to the first observed AP at rheobase, and number of APs, was performed by Mann-Whitney tests; exact *P*-values and the numbers of patched cells are provided in **Additional file 7**.

For Ca^{2+} imaging experiments of human SGN-like neurons, statistical comparisons were performed using unpaired Mann-Whitney tests. Human SGN-like neurons generated from three different passage numbers of hiPSCs were analyzed. Ca^{2+} signals were normalized to the mean baseline intensity.

All statistical analyses were conducted in GraphPad Prism 9.2.0 software. A $P<0.05$ was used to determine significance. The number of technical and biological replicates in each experiment are also listed in the figure captions.

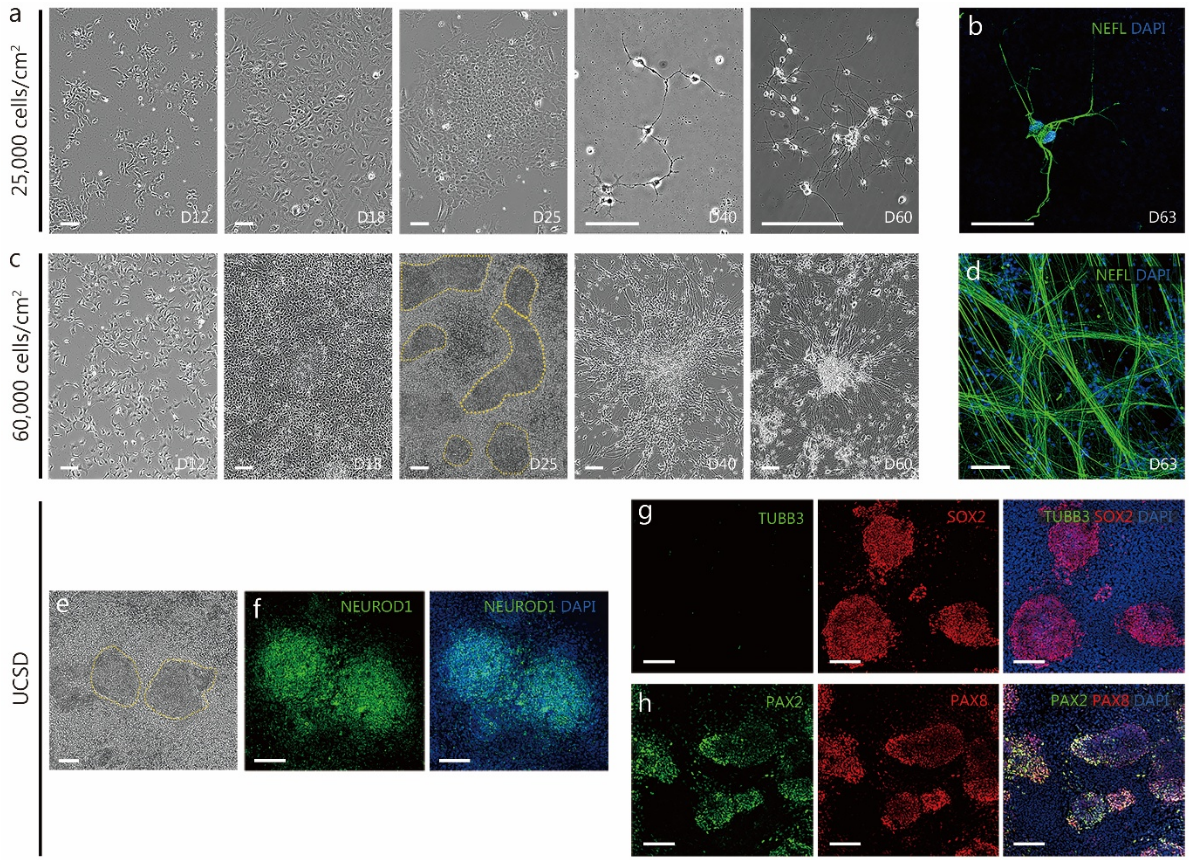

Fig. S1 Higher seeding density on D11 promotes the formation of ONP-like cells and subsequent neuronal development. **a**, **b** Low seeding density (25,000 cells/cm^2) of pre-placodal ectoderm and neural crest precursors (on D11) after sorting negatively affected subsequent differentiation into human SGN-like cells. The low seeding density also affected the length of neurites. The longest sizes of neurons stained with NEFL (**b**) from axon to dendrite were <200 µm on D63. SK8-A hiPSCs were used for this experiment. Scale bar=100 µm. **c**, **d** Higher seeding density (60,000 cells/cm^2) on D11 after sorting was the appropriate cell density to form vesicles of ONP-like cells (yellow dotted circles) on D25. NEFL staining (**d**) on D63 revealed clusters of human SGN-like neurons resembling mouse SGN explant cultures *in vitro* [8]. SK8-A hiPSCs were used for this experiment. Scale bar=100 µm. **e-h** Representative phase-contrast image on D25 (**e**) showing ONP-like cells (yellow dotted circles) arising from NGFR$^+$ cells seeded at 60,000 cells/cm^2 on D11. These ONP-like cells expressed the neuroblast marker NEUROD1 (**f**), but no TUBB3$^+$ neurons were observed (**g**). The D25 cultures also contained cells expressing otic lineage markers, such

as PAX2, PAX8 (**h**), and SOX2 (**g**). Scale bar=100 μm. All experiments were performed with at least 3 biological replicates and 3 technical replicates. D. Day; ONP. Otic neural progenitor; hiPSCs. Human induced pluripotent stem cells; SGN. Spiral ganglion neurons; NEFL. Neurofilament; NGFR. Nerve growth factor receptor; TUBB3. Tubulin beta 3 class III; PAX2. Paired box gene 2; SOX2. SRY-box transcription factor 2; DAPI. 4′,6-diamidino-2-phenylindole

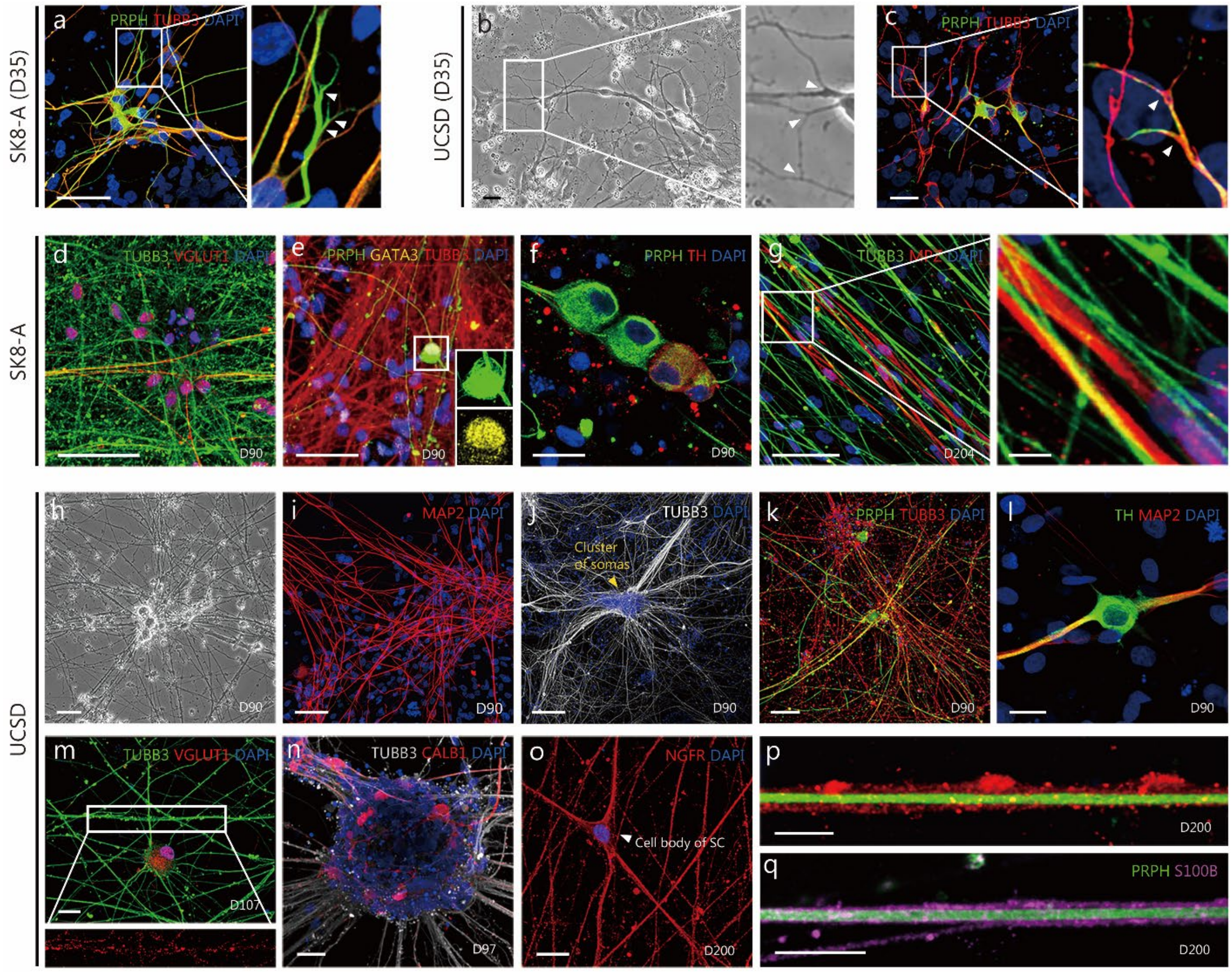

Fig. S2 Maturation and myelination of human SGN-like neurons. **a-c** Early-stage human SGN-like cells had two main sets of fibers extending from opposite sides of the cell body, including small sub-branches. On D35, approximately (89.4±9.5)% of human SGN-like neurons (**a**) expressed both PRPH and TUBB3. Scale bar=50 μm (**a**) and 20 μm (**b**, **c**). **d**, **m** Expression of the glutaminergic neuronal marker VGLUT1. Scale bar=50 μm (**d**) and 20 μm (**m**). **e**, **f** Maturing human SGN-like neurons displayed a bipolar morphology and separated into TUBB3$^+$ type I and PRPH$^+$, GATA3$^+$ (**e**), or TH$^+$ (**f**) type II subtypes. Scale bar=50 μm (**e**) and 20 μm (**f**). **g** MPZ expression in myelinating Schwann cell-like cells on D204 and its higher magnification view. Scale bar=50 μm. **h** Representative bright-field images of D90 human SGN-like neurons derived from the UCSD hiPSC line. Scale bar=50 μm. **i-l** Expression of pan-neuronal marker MAP2 (**i**), type I SGN marker TUBB3 (**j**), and type II markers PRPH (**k**), and TH (**l**) in D90 human SGN-like neurons. Scale bar=100 μm (**k**), 50 μm (**i**), 20 μm (**j**, **l**). **n** Expression of CALB1, a type Ia SGN marker,

was confirmed in mouse SGNs at postnatal stage 17 [9]. Scale bar=20 µm. **o-q** Wrapping of type II human SGN-like neural fibers by non-myelinating Schwann cell-like cells. Scale bar=20 µm (**o**), 10 µm (**q**), 5 µm (**p**). All experiments were performed using at least 3 biological replicates and 3 technical replicates. D. Day; SGN. Spiral ganglion neurons; SD. Standard deviation; PRPH. Peripheral neuronal marker peripherin; TUBB3. Tubulin beta 3 class III; hiPSC. Human-induced pluripotent stem cells; MAP2. Microtubule-associated protein 2; CALB1. Calbindin1; MPZ. Myelin protein zero; GATA3. GATA binding protein 3; TH. Tyrosine hydroxylase; VGLUT1. Vesicular glutamate transporter 1; NGFR. Nerve growth factor receptor; SC. Schwann cell; DAPI. 4′,6-diamidino-2-phenylindole

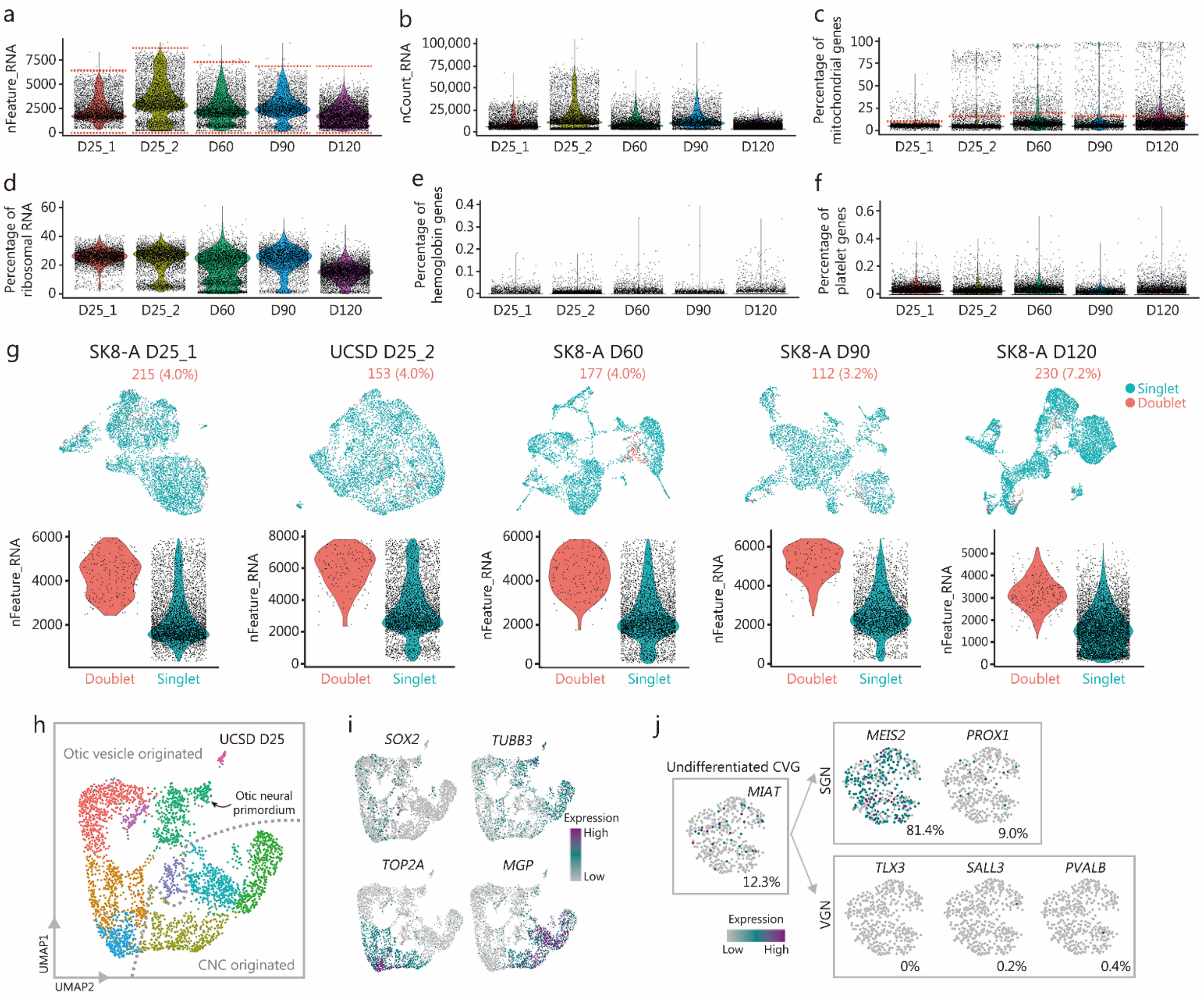

Fig. S3 Quality control and scRNA-seq data. **a** Violin plots indicating the number of genes detected in each cell (nFeature_RNA). The cut-off thresholds for D25_1 (SK8-A), D25_2 (UCSD), D60 (SK8-A), D90 (SK8-A), D120 (SK8-A) were <200 and >6000, <200 and >8000, <200 and >7000, <200 and >6500, and <200 and >6500, respectively. The red dashed line indicates cut-off fractions. **b** Violin plots showing the total number of molecules detected within a cell (nCount_RNA). **c** Violin plots showing the percentage of mitochondrial genes. Above, 10% in D25_1, 15% in D25_2, 20% in D60, 15% in D90, and 15% in D120 were filtered out. **d** Violin plots showing the percentage of ribosomal RNA. **e** Violin plots showing the percentage of hemoglobin genes. **f** Violin plots showing the percentage of platelet genes from each sample. **g** UMAP plots showing doublets detected by DoubletFinder, and violin plots showing the number

of genes detected in doublets. Doublets were removed, and only the predicted singlet cells were used for further analysis. **h** UMAP plots of UCSD D25 cells. **i** Feature plots displaying key gene markers for classifying cell subtypes in UCSD D25. **j** Feature plot visualizing the expression of undifferentiated CVG, SGN, and vestibular ganglion neuron (VGN) markers in the otic neural primordium cluster of UCSD D25 cells. scRNA-seq. Single-cell RNA sequencing; D. Day; CVG. Cochleovestibular ganglia; UMAP. Uniform manifold approximation and projection; SGN. Spiral ganglion neuron; VGN. Vestibular ganglion neuron; SOX2. SRY-box transcription factor 2; TUBB3. Tubulin beta 3 class III; TOP2A. DNA topoisomerase II alpha; MGP. Matrix gla protein; MIAT. Myocardial infarction associated transcript; MEIS2. Meis homeobox 2; PROX1. Prospero homeobox 1; TLX3. T cell leukemia homeobox 3; SALL3. Spalt like transcription factor 3; PVALB. Parvalbumin

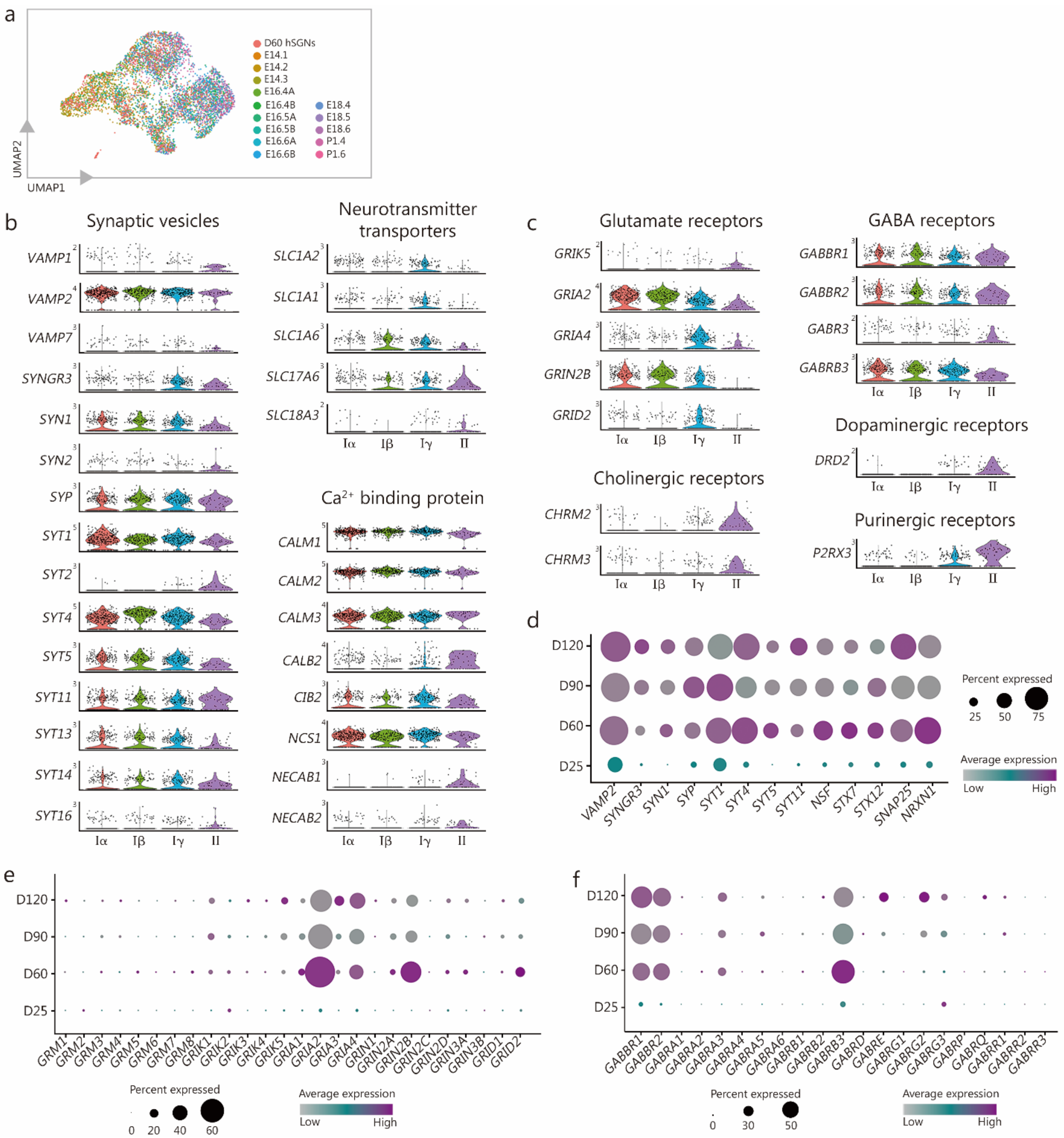

Fig. S4 Differential expression of genes related to neurotransmission. **a** UMAP plot of D60 human SGN-like neurons and mouse SGNs collected at E14–P1 [6]. **b** Violin plots illustrating differential expression of genes encoding synaptic vesicles, neurotransmitter transporters, and Ca^{2+} binding proteins in human SGN-like neurons at D60. **c** Violin plots representing expression of key genes related to glutamate, GABA, cholinergic, dopaminergic, and purinergic receptors in human SGN-like neurons at D60. **d** Expression levels of selected genes related to synaptic vesicles, neurotransmitter transports, and Ca^{2+} binding proteins

between D25 and D120 human SGN-like neurons. Expression levels of genes encoding glutamate (**e**) and GABA (**f**) receptors between D25 and D120 human SGN-like neurons. The number in the upper left corner represents log-normalized gene expression (log1p normalized UMI counts) per cell (**b, c**). UMAP. Uniform manifold approximation and projection; D. Day; SGN. Spiral ganglion neurons; Ca^{2+}. Calcium ion; GABA. Gmma-aminobutyric acid; E. Embryonic day; P. Postnatal day; VAMP. Vesicle associated membrane protein; SYNGR3. Synaptogyrin 3; SYN. Synapsin; SYP. Synaptophysin; SLC1A2. Solute carrier family 1 member 2; SLC1A1. Solute carrier family 1 member 1; SLC1A6. Solute carrier family 1 member 6; SLC17A6. Solute carrier family 17 member 6; SLC18A3. Solute carrier family 18 member 3; CALM1. Calmodulin; CALB2. Calbindin 2; CIB2. Calcium and integrin binding protein 2; NCS1. Neuronal calcium sensor 1; NECAB1. Neuronal calcium binding protein 1; NECAB2. Neuronal calcium binding protein 2; GRIK5. Glutamate ionotropic receptor kainate type subunit 5; GRIA2. Glutamate ionotropic receptor ampa type subunit 2; GRIA4. Glutamate ionotropic receptor ampa type subunit 4; GRIN2B. Glutamate ionotropic receptor nmda type subunit 2b; GRID2. Glutamate ionotropic receptor delta type subunit 2; CHRM2. Cholinergic receptor muscarinic 2; CHRM3. Cholinergic receptor muscarinic 3; GABBR1. Gamma-aminobutyric acid type b receptor subunit 1; GABBR2. Gamma-aminobutyric acid type b receptor subunit 2; GABR3. Gamma-aminobutyric acid type a receptor subunit gamma 3; GABRB3. Gamma-aminobutyric acid type a receptor subunit beta 3; DRD2. Dopamine receptor d2; P2RX3. Purinergic receptor p2x 3; NSF. N-ethylmaleimide sensitive factor; STX7. Syntaxin 7; STX12. Syntaxin 12; SNAP25. Synaptosome associated protein 25; NRXN1. Neurexin 1; GRM1. Glutamate metabotropic receptor 1; GRM2. Glutamate metabotropic receptor 2; GRM3. Glutamate metabotropic receptor 3; GRM4. Glutamate metabotropic receptor 4; GRM5. Glutamate metabotropic receptor 5; GRM6. Glutamate metabotropic receptor 6; GRM7. Glutamate metabotropic receptor 7; GRM8. Glutamate metabotropic receptor 8; GRIK1. Glutamate ionotropic receptor kainate type subunit 1; GRIK2. Glutamate ionotropic receptor kainate type subunit 2; GRIK3. Glutamate ionotropic receptor kainate type subunit 3; GRIK4. Glutamate ionotropic receptor kainate type subunit 4; GRIK5. Glutamate ionotropic

receptor kainate type subunit 5; GRIA1. Glutamate ionotropic receptor AMPA type subunit 1; GRIA2. Glutamate ionotropic receptor AMPA type subunit 2; GRIA3. Glutamate ionotropic receptor AMPA type subunit 3; GRIA4. Glutamate ionotropic receptor AMPA type subunit 4; GRIN1. Glutamate ionotropic receptor NMDA type subunit 1; GRIN2A. Glutamate ionotropic receptor NMDA type subunit 2a; GRIN2B. Glutamate ionotropic receptor NMDA type subunit 2b; GRIN2C. Glutamate ionotropic receptor NMDA type subunit 2c; GRIN2D. Glutamate ionotropic receptor NMDA type subunit 2d; GRIN3A. Glutamate ionotropic receptor NMDA type subunit 3a; GRIN3B. Glutamate ionotropic receptor NMDA type subunit 3b; GRID1. Glutamate ionotropic receptor delta type subunit 1; GRID2. Glutamate ionotropic receptor delta type subunit 2; GABBR1. Gamma aminobutyric acid type B receptor subunit 1; GABBR2. Gamma aminobutyric acid type B receptor subunit 2; GABRA1. Gamma aminobutyric acid type A receptor subunit alpha 1; GABRA2. Gamma aminobutyric acid type A receptor subunit alpha 2; GABRA3. Gamma aminobutyric acid type A receptor subunit alpha 3; GABRA4. Gamma aminobutyric acid type A receptor subunit alpha 4; GABRA5. Gamma aminobutyric acid type A receptor subunit alpha 5; GABRA6. Gamma aminobutyric acid type A receptor subunit alpha 6; GABRB1. Gamma aminobutyric acid type A receptor subunit beta 1; GABRB2. Gamma aminobutyric acid type A receptor subunit beta 2; GABRB3. Gamma aminobutyric acid type A receptor subunit beta 3; GABRD. Gamma aminobutyric acid type A receptor subunit delta; GABRE. Gamma aminobutyric acid type A receptor subunit epsilon; GABRG1. Gamma aminobutyric acid type A receptor subunit gamma 1; GABRG2. Gamma aminobutyric acid type A receptor subunit gamma 2; GABRG3. Gamma aminobutyric acid type A receptor subunit gamma 3; GABRP. Gamma aminobutyric acid type A receptor subunit pi; GABRQ. Gamma aminobutyric acid type A receptor subunit theta; GABRR1. Gamma aminobutyric acid type A receptor subunit rho 1; GABRR2. Gamma aminobutyric acid type A receptor subunit rho 2; GABRR3. Gamma aminobutyric acid type A receptor subunit rho 3

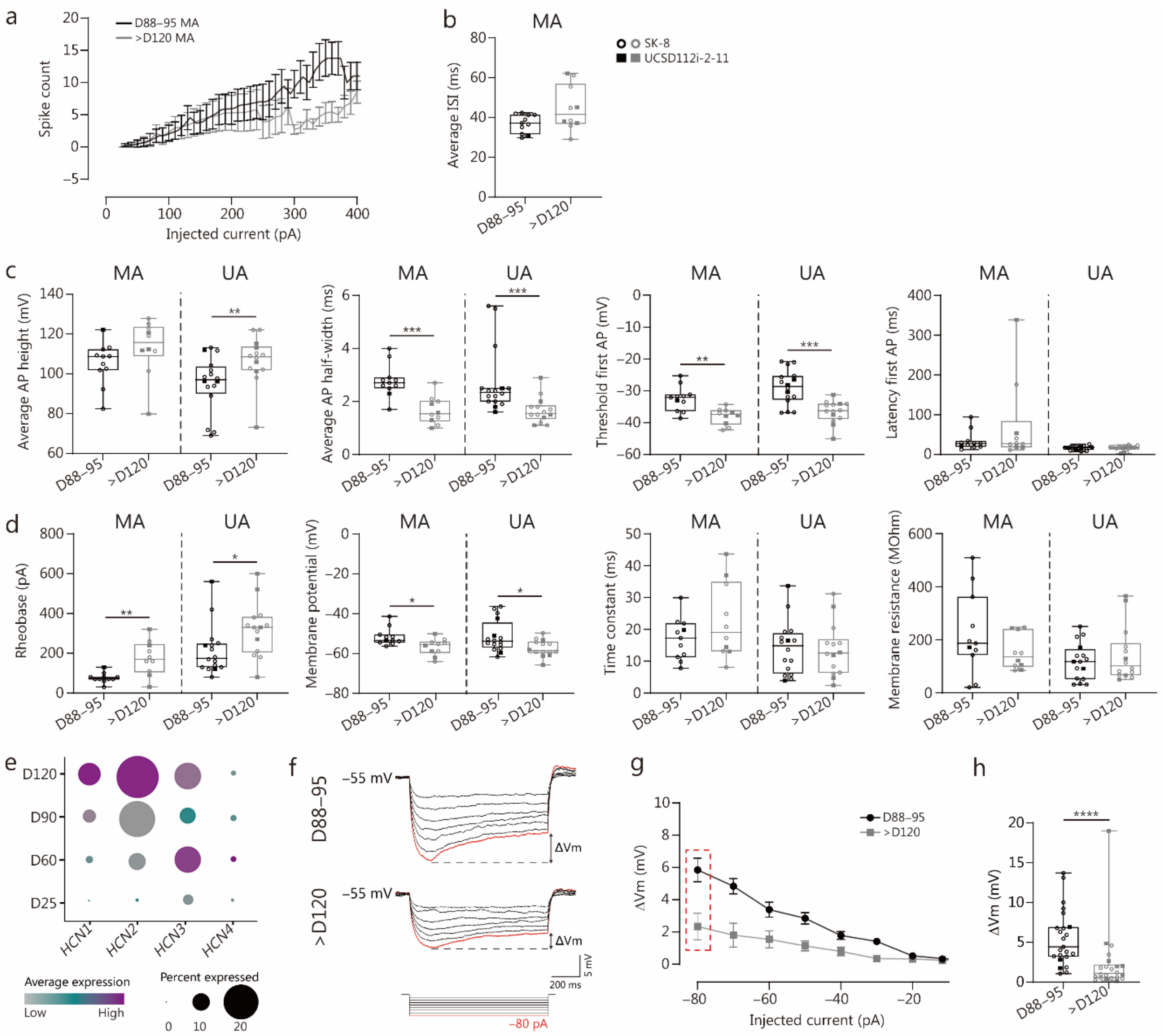

Fig. S5 Firing properties, AP parameters at rheobase, and voltage responses to hyperpolarizing steps in type I human SGN-like neurons. **a**, **b** Spike counts as a function of injected current (**a**) and average ISI (**b**) for MA type I human SGN-like neurons. D88–95, n=11; >D120, n=10. **c**, **d** AP properties analyzed at rheobase (**c**) and intrinsic properties (**d**) of type I human SGN-like neurons. D88–95: MA, n=11 and UA, n=16; >D120: MA, n=10 and UA, n=14. **e** mRNA expression levels of HCN channels. **f** Representative traces of current-clamp recordings in type I human SGN-like neurons in response to hyperpolarizing current steps. **g**, **h** Plot of the difference in membrane potential (ΔVm) between the peak of the hyperpolarizing response and the end of the current step as a function of hyperpolarizing current steps; red dashed square indicates average ΔVm at the -80 pA step, for D88–95 and >D120 (**g**). Comparison of

the average ΔVm at the -80 pA step for D88–95 and >D120 (**h**). D88–95, *n*=23; >D120, *n*=23. $^{*}P$ <0.05, $^{**}P$<0.01, $^{***}P$<0.001, $^{****}P$<0.0001. Error bars refer to the standard error of the mean (SEM) in all panels. AP. Action potential; SGN. Spiral ganglion neurons; ISI. Inter-spike interval; MA. Multi-spike accommodating neurons; D. Day; UA. Unitary-spike accommodating neurons; HCN. Hyperpolarization-activated cyclic nucleotide-gated

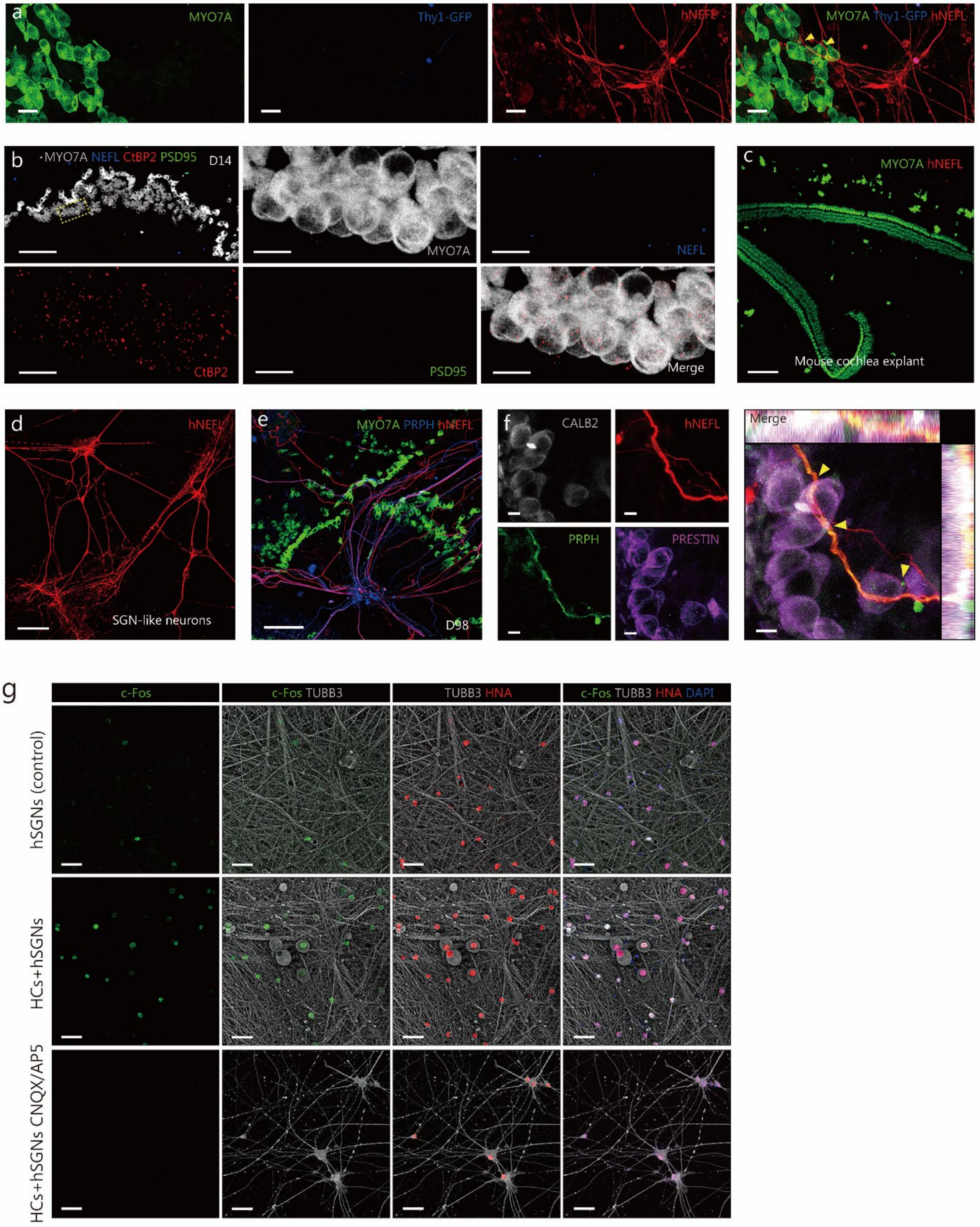

Fig. S6 Co-culture of hiPSC-derived human SGN-like neurons and mouse denervated hair cells. **a** Denervated hair cells from Thy1-GFP BL6 mice were used for co-culture with D44 human SGN-like neurons. No neurons expressing Thy1-GFP connected with hair cells. Scale bar=20 µm. **b** Absence of

neurite (NEFL$^+$) and PSD95$^+$ markers in denervated hair cells after 14 d of *in vitro* culture. The yellow inset shows higher magnification of mouse denervated hair cells expressing hair cell (MYO7A$^+$) and presynaptic (CtBP2$^+$) markers. Scale bar=10 µm. **c** Lack of hNEFL (a specific antibody for human neurofilament) expression in mouse cochlea explants. Scale bar=100 µm. **d** Positive detection of hNEFL in human SGN-like neurons. Scale bar=200 µm. **e** Overview of co-culture showing physical contact between hair cells and both type I and type II human SGN-like neurons. Scale bar=100 µm. **f** Contact between type II human SGN-like neurons (PRPH$^+$) and outer hair cells (PRESTIN$^+$) in co-culture. All inner and outer hair cells expressed CALB2. Scale bar=5 µm. **g** Immunostained images showing c-Fos expression in co-cultures of P4 mouse hair cells and D251 human SGN-like neurons. Human cells were specifically identified using HNA. Scale bar=50 µm. All experiments were performed using at least 3 biological replicates. CBA/CaJ mice were used for experiments in panels **b**, **c**, **e**, and **f**, while NOD/SCID mice were used for panel **g**. hiPSC. Human-induced pluripotent stem cells; SGN. Spiral ganglion neurons; D. Day; NEFL. Neurofilament; PSD95. Post-synaptic protein; PRPH. Peripheral neuronal marker peripherin; HNA. Human nuclear antigen; CALB2. Calbindin 2; MYO7A. Myosin VIIA; CtBP2. C-terminal binding protein 2; PSD95. Postsynaptic density protein 95; PRESTIN. Solute carrier family 26 member 5; TUBB3. Tubulin beta 3 class III

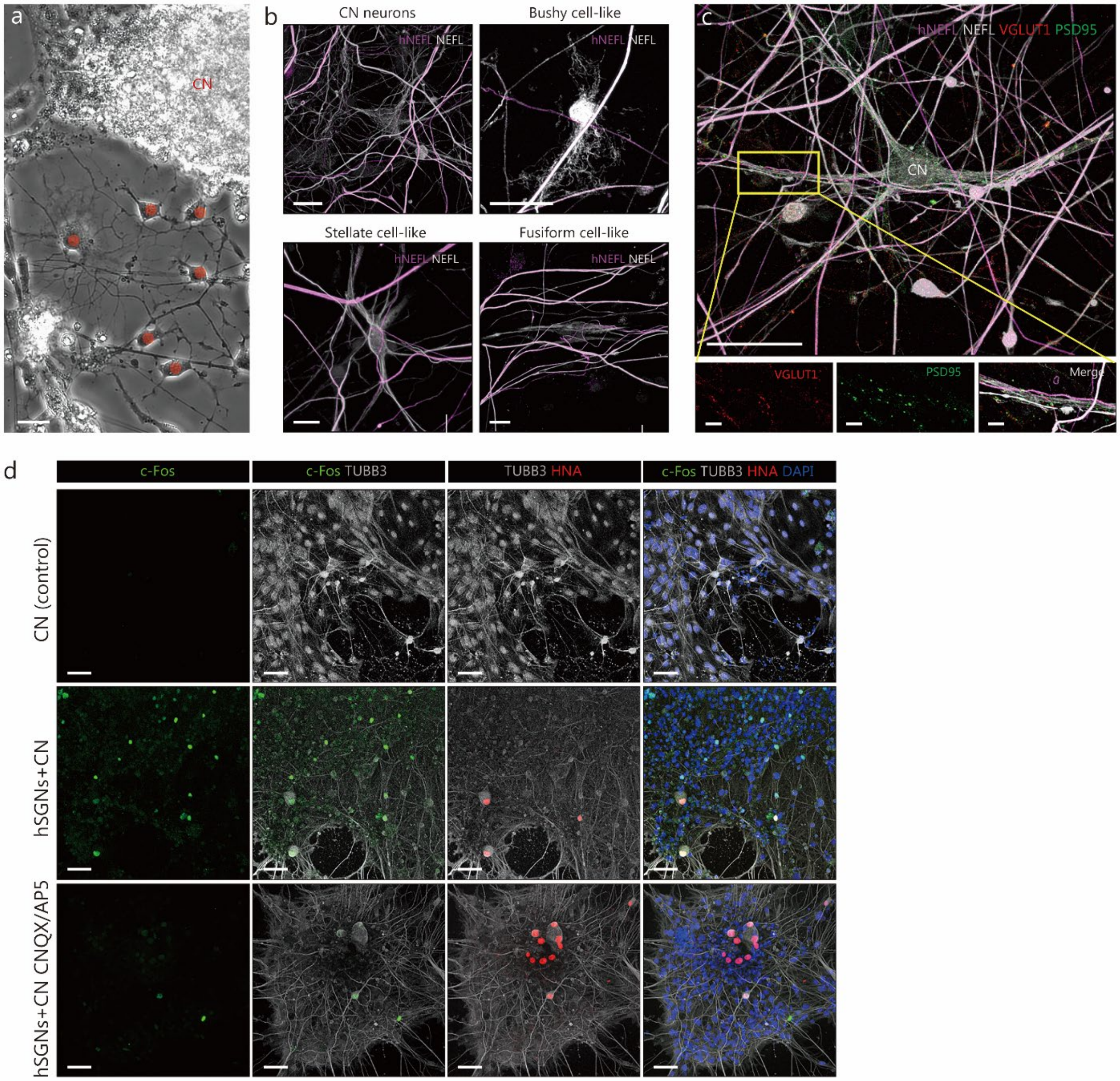

Fig. S7 Co-culture of hiPSC-derived human SGN-like neurons and mouse CN neurons. **a** Bright-field images of P5 CN tissue with CN neurons (red dots) emerging from the CN explant. Scale bar=20 µm. **b** Co-culture of D73 human SGN-like neurons (hNEFL^{+}, NEFL^{+}) with different types of mouse CN neurons (hNEFL^{-}, NEFL^{+}) from an 8-week-old mouse. Scale bar=20 µm. **c** Synaptic connections between mouse CN neurons (NEFL^{+}/hNEFL^{-}) from 8-week-old mice and D73 human SGN-like neurons (hNEFL^{+}) after 28 d in co-culture. Scale bar=50 µm. **d** Representative immunostained images showing c-Fos expression in co-cultures of D81 human SGN-like neurons with P4 CN neurons. Scale bar=50 µm. All experiments were performed using at least 3 biological replicates. CBA/CaJ mice were used for panels **a-c**, and

NOD/SCID mice were used for panel **d**. hiPSCs. Human-induced pluripotent stem cells; CN. Cochlear nucleus; D. Day; SGN. Spiral ganglion neurons; NEFL. Neurofilament; P. Postnatal day

References

1. Jeong M, Ocwieja KE, Han D, Wackym PA, Zhang Y, Brown A, *et al*. Direct SARS-CoV-2 infection of the human inner ear may underlie COVID-19-associated audiovestibular dysfunction. Commun Med. 2021;1(1):44.
2. McGinnis CS, Murrow LM, Gartner ZJ. DoubletFinder: doublet detection in single-cell RNA sequencing data using artificial nearest neighbors. Cell Syst. 2019;8(4):329-37.e4.
3. Luecken MD, Buttner M, Chaichoompu K, Danese A, Interlandi M, Mueller MF, *et al*. Benchmarking atlas-level data integration in single-cell genomics. Nat Methods. 2022;19(1):41-50.
4. Sun Y, Wang L, Zhu T, Wu B, Wang G, Luo Z, *et al*. Single-cell transcriptomic landscapes of the otic neuronal lineage at multiple early embryonic ages. Cell Rep. 2022;38(12):110542.
5. Sanders TR, Kelley MW. Specification of neuronal subtypes in the spiral ganglion begins prior to birth in the mouse. Proc Natl Acad Sci U S A. 2022;119(48):e2203935119.
6. Landegger LD, Dilwali S, Stankovic KM. Neonatal murine cochlear explant technique as an *in vitro* screening tool in hearing research. J Vis Exp. 2017;124:55704.
7. Parker M, Brugeaud A, Edge ASB. Primary culture and plasmid electroporation of the murine organ of corti. J Vis Exp. 2010(36):1685.
8. Meas SJ, Nishimura K, Scheibinger M, Dabdoub A. *In vitro* methods to cultivate spiral ganglion cells, and purification of cellular subtypes for induced neuronal reprogramming. Front Neurosci. 2018;12:822.
9. Petitpre C, Wu H, Sharma A, Tokarska A, Fontanet P, Wang Y, *et al*. Neuronal heterogeneity and stereotyped connectivity in the auditory afferent system. Nat Commun. 2018;9(1):3691.
